# SNX-3 mediates retromer-independent tubular endosomal recycling by opposing EEA-1-facilitated trafficking

**DOI:** 10.1371/journal.pgen.1009607

**Published:** 2021-06-03

**Authors:** Yangli Tian, Qiaoju Kang, Xuemeng Shi, Yuan Wang, Nali Zhang, Huan Ye, Qifeng Xu, Tao Xu, Rongying Zhang

**Affiliations:** 1 Key Laboratory of Molecular Biophysics of the Ministry of Education, College of Life Science and Technology, Huazhong University of Science and Technology, Wuhan, Hubei, China; 2 National Laboratory of Biomacromolecules, Institute of Biophysics, Chinese Academy of Sciences, Beijing, China; University of Massachusetts Medical School, UNITED STATES

## Abstract

Early endosomes are the sorting hub on the endocytic pathway, wherein sorting nexins (SNXs) play important roles for formation of the distinct membranous microdomains with different sorting functions. Tubular endosomes mediate the recycling of clathrin-independent endocytic (CIE) cargoes back toward the plasma membrane. However, the molecular mechanism underlying the tubule formation is still poorly understood. Here we screened the effect on the ARF-6-associated CIE recycling endosomal tubules for all the SNX members in *Caenorhabditis elegans* (*C*. *elegans*). We identified SNX-3 as an essential factor for generation of the recycling tubules. The loss of SNX-3 abolishes the interconnected tubules in the intestine of *C*. *elegans*. Consequently, the surface and total protein levels of the recycling CIE protein hTAC are strongly decreased. Unexpectedly, depletion of the retromer components VPS-26/-29/-35 has no similar effect, implying that the retromer trimer is dispensable in this process. We determined that hTAC is captured by the ESCRT complex and transported into the lysosome for rapid degradation in *snx-3* mutants. Interestingly, EEA-1 is increasingly recruited on early endosomes and localized to the hTAC-containing structures in *snx-3* mutant intestines. We also showed that SNX3 and EEA1 compete with each other for binding to phosphatidylinositol-3-phosphate enriching early endosomes in Hela cells. Our data demonstrate for the first time that PX domain-only *C*. *elegans* SNX-3 organizes the tubular endosomes for efficient recycling and retrieves the CIE cargo away from the maturing sorting endosomes by competing with EEA-1 for binding to the early endosomes. However, our results call into question how SNX-3 couples the cargo capture and membrane remodeling in the absence of the retromer trimer complex.

## Author summary

Trafficking of internalized materials through the endolysosomal system is essential for the maintenance of homeostasis and signaling regulation in all eukaryotic cells. Early endosomes are the sorting hub on the endocytic pathway. After internalization, the plasma membrane lipid, proteins, and invading pathogens are delivered to early endosomes for further degradation in lysosomes or for retrieval to the plasma membrane or the *trans*-Golgi network for reuse. However, when, where and by what mechanism various cargo proteins are sorted from each other and into the different pathways largely remain to be explored. Here, we identified SNX-3, a PX-domain only sorting nexin family member, as a novel regulator for the tubular endosomes underlying recycling of a subset of CIE cargoes. Compared with EEA-1, the superior recruitment of SNX-3 at the CIE-derived subpopulation of endosomes is critical for preventing these endosomes from converging to the classical sorting endosomes and subsequently into the multivesicular endosomal pathway. We speculate that through a spatio-temporal interplay with the retromer, SNX-3 is involved in different recycling transport carriers. Our finding of SNX-3’s role in modulating the formation of tubular endosomes provides insight into the sorting and trafficking of CIE pathways.

## Introduction

Endocytosis and post-endocytic trafficking through the endolysosomal system are essential for the maintenance of homeostasis and signaling regulation in eukaryotic cells. After internalization, the plasma membrane (PM) lipids and proteins, as well as nutrients, are delivered to the early endosomes (EEs) or sorting endosomes (SEs). Here, cargo proteins are sorted for degradation in lysosomes or alternatively retrieved and recycled to the PM or the *trans*-Golgi network (TGN) for reuse [1,2]. There are two general types of endocytic pathways, the clathrin-dependent (CDE) and clathrin-independent endocytosis (CIE) [3]. As increasing PM proteins are found to enter cells by CIE pathways [4], CIE mechanisms are recognized to be fundamental for many physiological processes, such as immune surveillance, cell signaling, cell migration, and metastasis [5]. However, compared to the well-established CDE pathway, our understanding of the details of CIE and post-endocytic trafficking is particularly limited [6,7].

Several protein complexes are involved in the sorting of transmembrane cargoes into the recycling/retrograde transport pathway or the degradative multivesicular endosomal pathway (MVE). Endosomal complexes required for transport (ESCRTs) are implicated in the formation and budding of intraluminal vesicles (ILVs) to recognize and sort ubiquitinated membrane proteins that arrive via the endocytic pathway [8]. The sorting nexin family of proteins (SNXs) are implicated in receptor recycling to the PM or retrieval to TGN and are characterized by the presence of a conserved phox (PX) domain, which mediates interactions with endosomal phosphoinositides (mainly phosphatidylinositol 3-phosphate, PtdIns(3)P) [9,10]. The evolutionarily conserved retromer, comprising a vacuolar protein-sorting trimer of VPS26-VPS29-VPS35 that is proposed to regulate cargo selection, can form alternative protein-sorting complexes with different SNX members [9]. Recent studies have documented that the SNX-BAR dimer and retromer complex function in endosome-to-TGN retrieval of CI-MPR [11,12]. By contrast, SNX27-retromer is implicated to recycle a variety of cell surface proteins back to the PM, including GLUT1 and many important neurotransmitter receptors, such as AMPA receptor, serotonin-4a receptor, and the β2 adrenergic receptor [13]. Interestingly, although SNX3 consists only of a PX-domain, SNX3-retromer complex is formed and involved in the retrograde transport of cargo receptors including the Wnt sorting receptor Wntless, the divalent metal ion transporter Dmt1-II, and even CI-MPR [14–16]. Nevertheless, when, where and by what mechanism the CIE/CDE cargo proteins are sorted from each other and into the different pathways of degradation and recycling is poorly understood.

The entry and subsequent intracellular itinerary followed by various ARF6-associated CIE cargo proteins have been investigated using the cultured mammalian cells [3]. Usually the internalized CIE proteins such as the major histocompatibility complex class I (MHCI), the GPI-anchored protein CD59, and GLUT1 join the classical SEs containing the CDE cargo transferrin receptor (TfR) and the early endosomal antigen 1 (EEA1) soon after internalization, where they are sorted for recycling or degradation [7]. However, a subset of CIE proteins, like CD44, CD98, and CD147, enter cells with MHCI and directly join the recycling tubules, by-passing the merge with classical EEA1-positive SEs [4]. The existence of these divergent itineraries suggests that the CIE cargoes are sorted at different points along the endo-lysosomal pathway. Consequently, it remains controversial as to how many independent types of recycling transport carriers are formed from endosomes and what their relative relationship is. The α-chain of the human interleukin-2 receptor (IL-2Rα, also termed hTAC) is also a classical marker of ARF6-associated CIE pathway [17]. Using the *Caenorhabditis elegans* (*C*. *elegans*) intestinal integrated hTAC, Grant and his colleagues have identified a set of CIE specific recycling regulators, including RAB-10, EHBP-1 and ALX-1 [18–20]. We previously found that a component of the exocyst complex SEC-10, in concert with RAB-10 and the microtubule cytoskeleton, plays an important role in the formation of the interconnected endosomal tubules required for efficient recycling of hTAC in the *C*. *elegans* intestine [21]. However, there is still a far way to go to fully understand the mechanisms involved in remodeling/generation of the CIE-relevant tubular endosome membrane and how this process is coordinated with selective capture of recycling cargoes.

The present work explores the mechanisms underlying the sorting and trafficking of CIE cargoes within the endo-lysosomal system. We performed an imaging-based family-wide screening of SNX proteins and identified candidates whose dysfunction disrupted hTAC-containing tubular endosomes in the *C*. *elegans* intestine. SNX-3 was identified as a novel regulator of the tubular endosomes that mediates specific ARF-6-associated CIE recycling independent of the retromer complex. The loss of SNX-3 abolishes the ARF-6-associated CIE recycling endosomal tubules and results in misrouting of hTAC into the lysosome for degradation. Furthermore, we demonstrated a competitive relationship between SNX-3 and EEA-1 for association with PtdIns(3)P-enriched EEs. Accordingly, the loss of SNX-3 results in the merge of hTAC-laden endosomal intermediates with EEA-1-positive endosomes and ultimately entry into lysosomes. The identification of the role of SNX-3 in modulating the formation of the tubular endosomes and recycling of the ARF-6-associated CIE cargoes provides insight into the sorting and trafficking of CIE pathways.

## Results

### SNX-3 regulates the ARF-6-associated CIE recycling endosomal tubules

**Fig 1:**
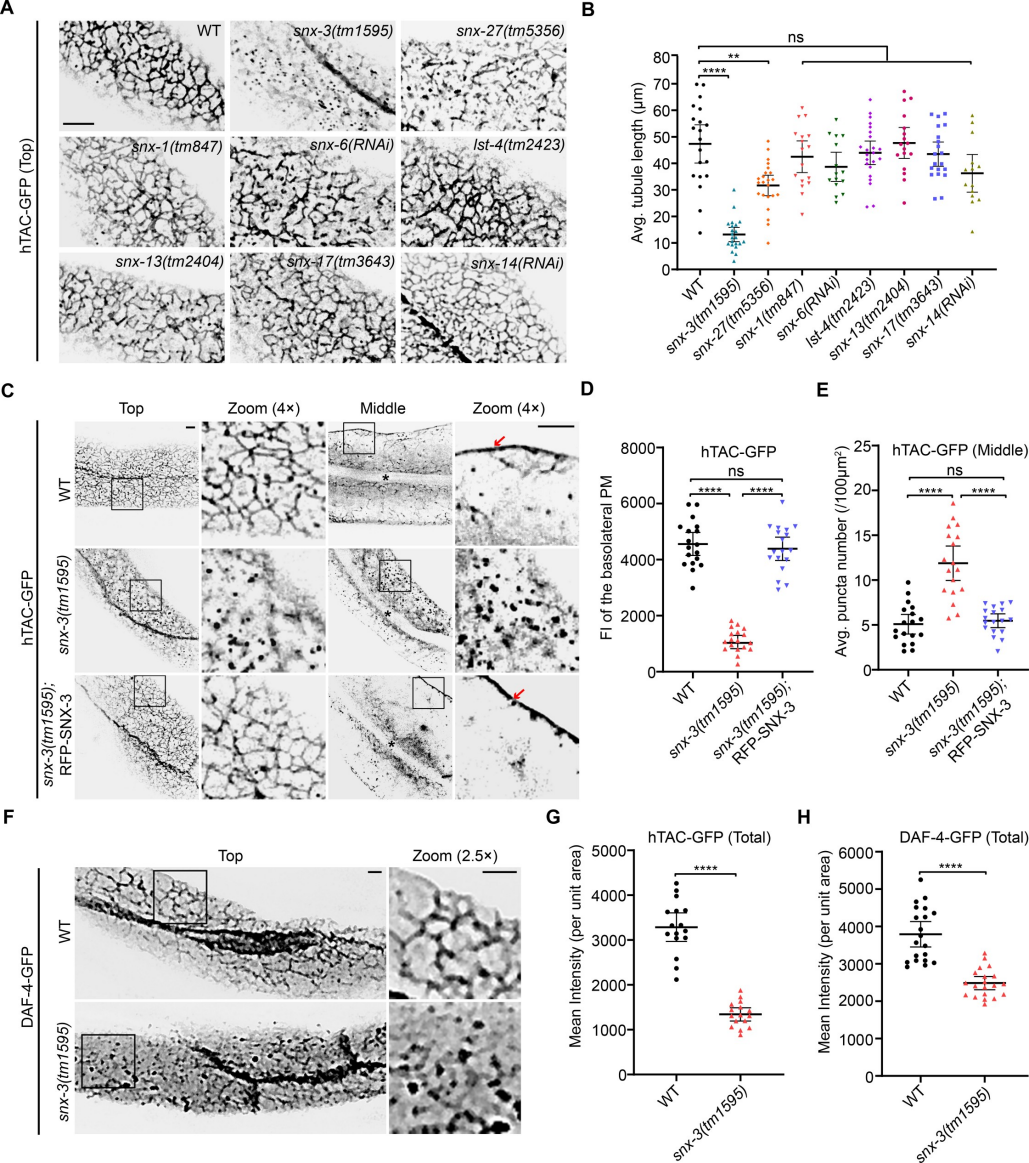
*C*. *elegans* SNX-3 is required for formation of clathrin-independent recycling endosomal tubules. (A) Confocal images provide the effect of various SNX mutant or RNAi treatment on the subplasmalemmal hTAC-GFP-containing tubular endosomes. (B) The average length of hTAC-positive tubules per 100 μm^2^ was calculated for intestines of each indicated genotype. Error bars are mean ± 95% CI ([ROI] = 20/22/23/17/15/23/17/20/14, n = 20/22/23/17/15/23/17/20/14 for WT/*snx-3(tm1595)*/*snx-27(tm5356)*/*snx-1(tm847)*/*snx-6(RNAi)*/*lst-4(tm2423)*/*snx-13(tm2404)*/*snx-17(tm3643)*/*snx-14(RNAi)*). ns, not significant; **P<0.01; ****P<0.0001 (Brown-Forsythe and Welch ANOVA with Dunnett T3 multiple comparison test to WT). (C) Micrographs showing the detailed distribution and architectures for intestinal hTAC-GFP signal. In *snx-3(tm1595)* mutants, subplasmalemmal hTAC-GFP-containing tubular endosomes (Top) were severely fragmented and punctate hTAC-GFP signals accumulated in the cytoplasm (Middle). Crossing of the *vha-6* driven RFP-SNX-3 MosSCI insertion strain into *snx-3(tm1595)* mutants restored the tubular profiles of hTAC-containing structures. The arrows indicate basolateral PM-associated hTAC-GFP. Asterisks depict the intestinal lumen. (D,E) The average fluorescence intensity (FI) of hTAC-GFP at the basolateral membrane (D) and the number of cytoplasmic hTAC-GFP puncta (E) were calculated for intestines of each indicated genotype. Region of interest [ROI] = 18; the number of animals analyzed n = 18 in groups of D and n = 12 in groups of E. Error bars are mean ± 95% CI. ns, not significant; ****P<0.0001 (Brown-Forsythe and Welch ANOVA with Dunnett T3 multiple comparison test). (F) Confocal images showing disruption of the tubular profiles of GFP-tagged endogenous cargo DAF-4 in *snx-3(tm1595)* mutants. (G,H) Quantification of the average total FI of hTAC-GFP (G) and DAF-4-GFP (H) for intestines of indicated genotypes. In *snx-3(tm1595)* mutants, both FIs decreased relative to the WT animals. In G, [ROI] = 16, n = 16; in H, [ROI] = 20, n = 20. Error bars are mean ± 95% CI. ****P<0.0001 (*t* test with Welch’s correction). Scale bars: 5 μm. Quantitative data are available in S1 File.

Searches of *C*. *elegans* genome databases identified eight *snx* genes, namely, *snx-1*, *snx-6*, *snx-3*, *snx-17*, *snx-27*, *lst-4*/*snx9*/*18*/*33*, *snx-13*, and *snx-14* [22,23]. The effect of the various SNXs on the hTAC-containing endosomal structures was investigated using confocal microscopy. In wild-type (WT) intestines, most hTAC-GFP signals were ARF-6-positive and displayed delicate tubule-vesicular network beneath the basolateral plasmalemma (top layer) (Figs S1A and 1A). A small number of puncta were in the cytoplasm (middle layer) [21,24]. More than 70% and about 33% of the hTAC tubules were fragmented in *snx-3(tm1595)* and *snx-27(tm5356)* mutants, respectively. The loss of the 6 other SNX members did not alter the tubule-vesicular network (Fig 1A and 1B). Analysis of the gene transcriptional expression pattern revealed that both *snx-3* and *snx-27* are ubiquitously expressed in multiple tissues of the adult *C*. *elegans* including the intestine (S2 Fig). As SNX-3 had more severe effects on the hTAC tubules than SNX-27 had, we focused on the role of SNX-3 in the formation of hTAC-containing CIE tubular endosomes.

Of note, in the *snx-3(tm1595)* null allele [14], the subplasmalemmal hTAC-containing endosomal tubules were fragmented into punctate structures, and the GFP fluorescence intensity (FI) decreased by about 75% (Fig 1C and 1D). The number of hTAC-GFP puncta in the cytosol was about 2.33-fold of that in the WT intestine (Fig 1C and 1E). DAF-4 (dauer formation-defective-4) is the *C*. *elegans* homolog of type II bone morphogenetic protein (BMP) receptor, another ARF-6-associated CIE recycling cargo protein [25]. DAF-4-GFP tubules underneath the basal PM were also changed to punctate in the *snx-3* mutant background (Fig 1F), as observed for hTAC. Moreover, the mean intensities of GFP-tagged hTAC and DAF-4 decreased by 59.1% and 34.4%, respectively, suggesting a reduction of the protein levels in *snx-3(tm1595)* mutants (Fig 1G and 1H). In contrast, the steady-state subcellular distribution of the classical CDE recycling cargo hTfR (human transferrin receptor) or YP170 (oocyte yolk protein) was unaltered in the *snx-3(tm1595)* mutants, indicating that SNX-3 is dispensable for the post-endocytic trafficking of these CDE proteins in *C*. *elegans* (S1B Fig).

Next, we investigated whether the disrupted CIE tubular endosomes in *snx-3(tm1595)* mutants arises from a cell autonomous activity of SNX-3 within the intestine. Full-length SNX-3 was tagged at its N-terminus with RFP and expressed under an intestine-specific promoter (*vha-6*). A single copy insertion line was generated to minimize the potential ectopic localization of SNX-3 protein induced by the overexpression [26] (S3 Fig). Crossing this line into the *snx-3(tm1595)* null allele fully rescued the defect in the hTAC-containing tubular endosomes (Fig 1C–1E). We then explored the impact of the different regions of SNX-3 on its subcellular localization and the generation of the CIE tubular endosomes (S4 Fig). SNX-3 missing the N-terminal region (RFP-SNX-3-ΔN) restored the tubular profile of hTAC-containing structures in *snx-3(tm1595)* mutants in a similar fashion than full-length SNX-3 did. However, SNX-3 with a point mutation [RFP-SNX-3(Y71A)] defective in the binding of the PX domain to PtdIns(3)P or SNX-3 missing the C-terminal region (RFP-SNX-3-ΔC) was distributed across the cytosol and failed to rescue the fragmented hTAC-containing structures [27]. Collectively, these results strongly indicated that SNX-3 plays a cell-autonomous role in regulating the intestinal hTAC-containing CIE recycling endosomal tubules and that the C-terminal region and the PtdIns(3)P-binding PX domain are required for SNX-3 function.

### The CIE cargo protein hTAC is lysosomally degraded in *snx-3* mutants

**Fig 2:**
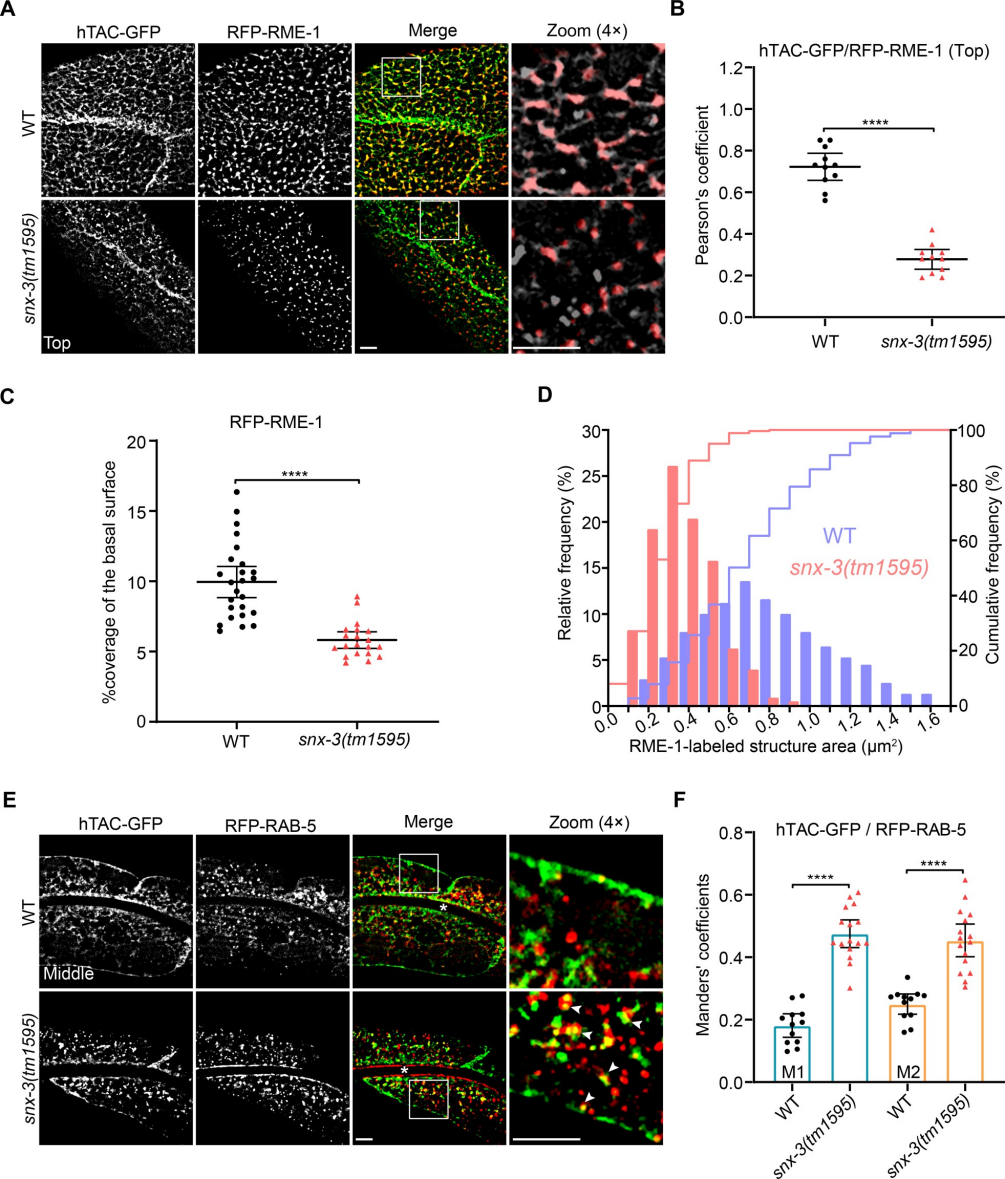
Loss of SNX-3 leads to misrouting of hTAC away from the recycling pathway. (A) Micrographs taken with a 150× objective showing the detailed architecture and location of RFP-RME-1-labeled basolateral recycling endosomes with respect to hTAC-GFP-containing carriers. In *snx-3 (tm1595)* mutants, the rod-like profiles of RFP-RME-1 prominently changed to spherical ones and shrank in size. (B) Quantification of the Pearson’s correlation coefficients for hTAC-GFP and RFP-RME-1 signals as depicted in A, error bars are mean ± 95% CI ([ROI] = 11, n = 11). ****P<0.0001 (Student’s *t* test). (C) Coverage percentage of the basal surface by RFP-RME-1, as the magnified regions shown in A, was quantified. WT: [ROI] = 25, n = 6; *snx-3(tm1595)*: [ROI] = 20, n = 5. Error bars are mean ± 95% CI. ****P<0.0001 (*t* test with Welch’s correction). (D) Effect of loss of SNX-3 on the size of RME-1-labeled structures. Histogram and cumulative distributions showed prominent decrease in the areas of RFP-RME-1 structures in *snx-3(tm1595)* mutants, analyzed for 253/262 RME-1-positive structures from 6/5 animals (WT/*snx-3(tm1595)*). (E) Confocal images showing colocalization between hTAC-GFP and EEs labeled by RFP-RAB-5. Compared with the WT animals, the overlap level of hTAC-GFP with RFP-RAB-5 is significantly increased in *snx-3(tm1595)* mutants. The arrowheads indicate positive overlap. Asterisks depict the intestine lumen. (F) Manders’ coefficients for hTAC-GFP and RFP-RAB-5 as depicted in E were calculated, error bars are mean ± 95% CI (WT: [ROI] = 12, n = 9; *snx-3(tm1595)*: [ROI] = 16, n = 10). ****P<0.0001 (Student’s *t* test for M1; *t* test with Welch’s correction for M2). M1: green pixels overlapping red; M2: red pixels overlapping green. Scale bars: 5 μm. Quantitative data are available in S1 File.

The preceding experiments showed a loss of hTAC and DAF-4 from the intestinal cell surface and probably at the whole-cell level in *snx-3(tm1595)* mutant animals. To determine whether they were lost owing to lysosomal degradation, we first assessed the localization of hTAC-GFP with respect to RME-1 in *snx-3(tm1595)* mutants. RME-1 is the only member of Eps15 homology (EH) domain-containing proteins (EHDs) in *C*. *elegans*. It specifically localizes to basolateral recycling endosomes and promotes the recycling of proteins from endosomes back to the cell surface [21,28,29]. In WT intestines, most of the subplasmalemmal hTAC tubules colocalized with RME-1-labeled structures, indicating efficient recycling. In *snx-3(tm1595)* animals, however, the overlap of the residual subplasmalemmal hTAC-GFP punctae with RME-1-labeled structures decreased by *ca*. 61.1% [Pearson’s coefficient of 0.72 for WT and 0.28 for *snx-3(tm1595)*] (Fig 2A and 2B). Moreover, the loss of SNX-3 caused a prominent decrease of the rod-like RME-1-positive structures, and the coverage of the basal surface by RME-1-positive elements decreased from *ca*. 9.9% to 5.8% (Fig 2C); in line with which, the contour areas of almost all the RME-1-positive structures in *snx-3(tm1595)* mutants were less than 0.7 μm^2^, whereas at least 49.8% of the RME-1-positive structures displayed contour areas exceeding 0.7 μm^2^ in WT animals (Fig 2D). The distribution of the peak area of RME-1-positive structures was significantly shifted to the left, indicating a reduction of the hTAC-containing REs in *snx-3(tm1595)* mutants. The fraction of cytoplasmic hTAC-GFP residing on RAB-5-labeled EEs was increased, as revealed by the increase of the Manders’ coefficient M1 from 0.18 for WT to 0.48 for *snx-3(tm1595)* animals (Fig 2E and 2F). Meanwhile, the fraction of RAB-5-EEs positive for hTAC-GFP was also increased, with a Manders’ coefficient M2 increasing from 0.25 for WT to 0.45 for *snx-3(tm1595)* animals (Fig 2F). Collectively, these results suggested that the trafficking of hTAC from EEs to REs was severely impaired by the loss of SNX-3.

**Fig 3:**
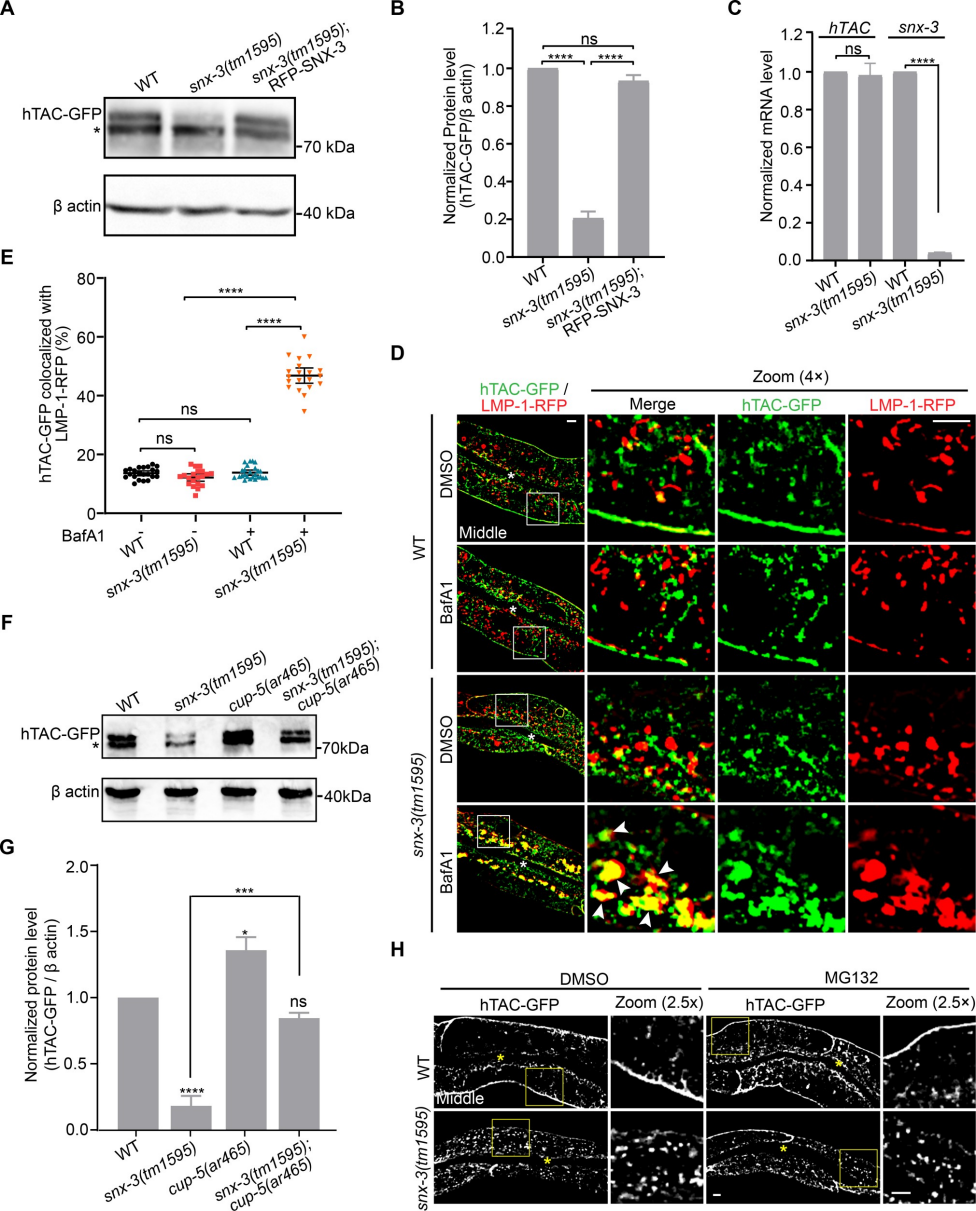
hTAC is efficiently degraded in the intestinal lysosome of *snx-3(tm1595)* mutants. (A,B) Western blot showed the hTAC-GFP level in *snx-3(tm1595)* mutants was significantly reduced compared with WT animals, and the reduction of hTAC-GFP level was reversed by re-expression of RFP-SNX-3 driven by intestine specific *vha-6* promoter. The lower band depicted by the asterisk is thought to represent non-specific antibody binding since it was observed in each lanes. Data are mean ± SEM from three independent experiments. ns, not significant; ****P<0.0001 (Student’s *t* test). (C) qRT-PCR analysis shows no change in the amount of *hTAC* mRNA in *snx-3(tm1595)* mutant animals. Data are mean ± SEM from four independent experiments. ns, not significant; ****P<0.0001 (Student’s *t* test). (D) Micrographs showing the colocalization of hTAC-GFP with LMP-1-RFP-labeled intestinal LEs and lysosomes in *snx-3(tm1595)* mutants pretreated with BafA1. In WT animals, no obvious colocalization of hTAC-GFP and LMP-1-RFP signals are observed either in the absence or presence of BafA1. By contrast, BafA1 treatment leads to accumulation of hTAC-GFP that prominently overlapped with LMP-1-RFP signals in *snx-3(tm1595)* mutants, indicating hTAC is efficiently degraded in the lysosome of *snx-3(tm1595)* mutants. (E) Colocalization of hTAC-GFP with LMP-1-RFP was calculated for each condition as depicted in D. [ROI] = 22/20/25/20, n = 22/9/25/11 for WT(-)/*snx-3(tm1595)*(-)/WT(+)/*snx-3(tm1595)*(+). Error bars are mean ± 95% CI. ns, not significant; ****P<0.0001 (Kruskal-Wallis test with Dunn’s *post hoc* test for multiple comparison). (F) Western blot showing the protein level of hTAC-GFP in WT, *snx-3(tm1595)* mutant, *cup-5(ar465)* mutant and *snx-3(tm1595); cup-5(ar465)* double mutant animals, respectively. Blockage of lysosomal function arising from loss of CUP-5 significantly reversed the reduced hTAC-GFP level in *snx-3(tm1595)* mutants. The asterisk depicts non-specific band. (G) Densitometry analysis of three independent experiments as in F is shown with mean ± SEM. ns, no significance, ****P<0.0001, ***P<0.001, *P<0.05 (one-way ANOVA followed by a Tukey’s *post hoc* test). (H) Micrographs showing MG132 treatment to block proteasomal degradation has no effect on hTAC-GFP in *snx-3(tm1595)* mutants. In D and H, the arrowheads indicate positive overlap. Asterisks depict the intestine lumen. Scale bars: 5 μm. Quantitative data are available in S1 File.

We then examined the effect of loss of SNX-3 on the steady-state total hTAC protein level. Western blot and the qRT-PCR results showed that the hTAC-GFP level significantly decreased in *snx-3(tm1595)* mutants, whereas the mRNA amounts were comparable to that of WT animals (Fig 3A–3C). Crossing the *vha-6*-driven single-copy RFP-SNX-3 transgene into the *snx-3(tm1595)* null background fully rescued the decreased hTAC-GFP protein (Fig 3A–3C). These results prompted us to suspect that the loss of SNX-3 result in the misrouting of the CIE cargo hTAC-GFP away from the recycling pathway and toward the degradation pathway. To test this hypothesis, we examined the subcellular localization of hTAC-GFP with respect to LMP-1-RFP-positive lysosomes [30]. Unexpectedly, no overlap of the cytosolic punctate hTAC-GFP with LMP-1-RFP was observed for either WT or the *snx-3(tm1595)* animals (percentage of colocalization: 13.6% and 12.2%, respectively) (Fig 3D and 3E). The apparent lack of location in lysosomes might be caused by a highly efficient degradation of hTAC-GFP. Therefore, bafilomycin A1 (BafA1) was injected into the body cavity, which may then penetrate into the intestine, to inhibit the lysosomal acidification [31,32]. The subcellular localization of hTAC-GFP was examined 6 h after injection (Fig 3D and 3E). As expected from the recycling route taken by hTAC-GFP, no colocalization between hTAC-GFP and LMP-1-RFP was detected in WT animals treated with BafA1. In contrast, many hTAC-GFP patches accumulated and overlapped with LMP-1-positive structures in the *snx-3* mutant intestines treated with BafA1 (colocalization coefficient of 46.8%) (Fig 3D and 3E). A similar distribution was also obtained for DAF-4-GFP (S5A Fig). To ascertain that hTAC-GFP was degraded in lysosomes of *snx-3(tm1595)* mutants, the function of lysosomal degradation was inhibited, and the steady-state protein level of hTAC-GFP in *snx-3(tm1595)* mutants was examined by immunoblot. CUP-5 is the *C*. *elegans* functional orthologue of mammalian Ca^2+^ channel mucolipin, which localizes at mature and nascent lysosomal membranes [33]. In *cup-5* mutants, the endocytosed material is not degraded and accumulates in large vacuoles [34]. The hTAC-GFP protein level was slightly higher in *cup-5(ar465)* single mutants than that in WT animals, suggesting that most hTAC was being recycled, whereas limited hTAC amount entered the lysosome for degradation. In contrast, the total hTAC-GFP level was prominently increased in *snx-3(tm1595); cup-5(ar465)* double mutants compared to the hTAC-GFP amount in the *snx-3(tm1595)* single mutant animals and was almost restored back to the WT level (Fig 3F and 3G). Altogether, we offered compelling evidence that loss of SNX-3 leads to mis-trafficking of a subset of ARF-6-associated CIE recycling cargoes into lysosomes where they are efficiently degraded.

We also examined the possibility that the decreased hTAC-GFP protein level in *snx-3(tm1595)* mutants is due to decomposition of the misfolded protein in the proteasome. MG132 was injected into the body cavity of *C*. *elegan*s to inhibit the intestinal proteasome [35]. In contrast to BafA1 treatment, no obvious aggregation of hTAC-GFP was observed in *snx-3(tm1595)* mutants after treatment of MG132 for 6 h (Fig 3H), indicating that the decrease of hTAC-GFP amount in *snx-3(tm1595)* was not caused by proteasomal degradation.

### hTAC traffics via the ESCRT pathway for lysosomal degradation in *snx-3* mutants

**Fig 4:**
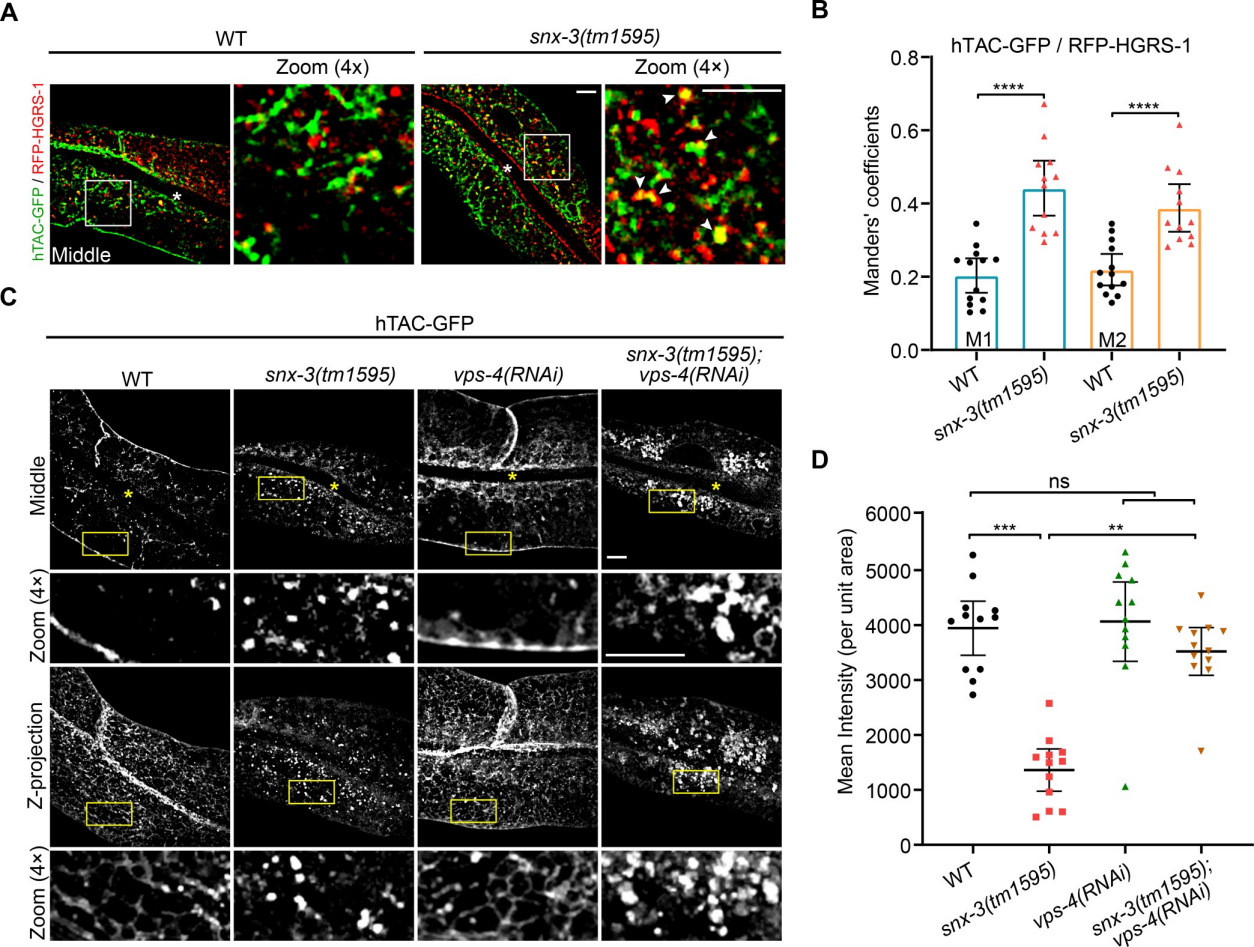
hTAC is captured by the ESCRT complex in *snx-3(tm1595)* mutants. (A) Confocal images showing the overlap of RFP-HGRS-1 with hTAC-GFP is increased in *snx-3(tm1595)* mutants. (B) Manders’ colocalization coefficients for hTAC-GFP and RFP-HGRS-1 as depicted in A were calculated, error bars are mean ± 95% CI (WT: [ROI] = 13, n = 9; *snx-3(tm1595)*: [ROI] = 12, n = 7). ****P<0.0001 (Student’s *t* test). M1: green pixels overlapping red; M2: red pixels overlapping green. (C) Confocal images showing the distribution of hTAC-GFP in intestines of *snx-3(tm1595)* mutant, *vps-4 RNAi*-treated and *snx-3(tm1595); vps-4(RNAi)* double mutant animals. hTAC-GFP accumulated into clusters of vacuoles in the intestine of *snx-3(tm1595); vps-4(RNAi)* double mutants. (D) The average FIs of hTAC-GFP depicted as Z-projection images in C were calculated. Error bars are mean ± 95% CI ([ROI] = 12, n = 12). ns, not significant; ***P<0.001, **P<0.01 (Kruskal-Wallis test with Dunn’s *post hoc* test for multiple comparison). In A and C, the arrowheads indicate positive overlap. Asterisks depict the intestine lumen. Scale bars: 5 μm. Quantitative data are available in S1 File.

Two opposing cargo sorting systems are located at the dynamic SEs. The ESCRT complex serves to recognize and sort ubiquitinated endosomal proteins for degradation. It is also involved in deforming the endosomal-limiting membrane inward to generate MVBs [36,37]. By contrast, the retromer complex associates with alternative SNXs and mediates the sorting and transport of various transmembrane proteins destined either to the Golgi or directly to the PM [15,38]. To clarify how the shunted cargo molecule is delivered to lysosomes, we used transgenes expressing fluorescently tagged HGRS-1/Hrs to mark the degradative ESCRT domain [8,37] and examined whether hTAC or DAF-4 proteins pass through ESCRT-positive structures to lysosomes. The Manders’ coefficients for the cytoplasmic hTAC-GFP and RFP-HGRS-1 revealed an increased colocalization of them in *snx-3(tm1595)* mutants; specifically, M1 increased from 0.20 in WT to 0.44 in *snx-3(tm1595)* mutants and M2 increased from 0.22 to 0.39 (Fig 4A and 4B). Similarly, M1 and M2 Manders’ coefficients for DAF-4-GFP and RFP-HGRS-1 increased significantly in *snx-3(tm1595)* mutants compared to those of WT animals (S5C and S5D Fig). The results indicated that the shunted CIE cargoes enter the lysosome via the ESCRT pathway.

VPS4 is a component of the AAA ATPase and is involved in the final step of MVB vesicle formation by dissociating or driving sequential polymerization of ESCRT-III [39,40]. To verify that the shunted cargo molecule is captured by the ESCRT complex for lysosomal degradation, the subcellular distribution of hTAC-GFP in *snx-3(tm1595); vps-4(RNAi)* double mutants was examined and compared with the individual single mutant (Fig 4C and 4D). After treatment with RNAi against *vps-4*, hTAC-GFP still mainly localized beneath the basolateral plasmalemma in tubular-vesicles with relative less puncta within the cytoplasma, as observed in control animals. Double depletion of SNX-3 and VPS-4, however, produced prominent clustering of hTAC-GFP-containing vacuoles in the cytoplasm (Fig 4C). In contrast to the *snx-3(tm1595)* mutants, the average total intensity of hTAC-GFP in the *snx-3(tm1595); vps-4(RNAi)* double mutants was comparable to those in WT or *vps-4*-RNAi treated animals (Fig 4D). These data suggest that even though the loss of SNX-3 causes hTAC to deviate from the recycling pathway, the depletion of VPS-4 further leads to the blockage of downhill flow of hTAC into the lysosome, resulting in clustered hTAC-containing structures. Taken together, these results demonstrated that the shunted CIE recycling cargo is mediated by ESCRT into the lysosome for degradation in *snx-3(tm1595)* mutants.

### The retromer trimer is dispensable for SNX-3-facilitated CIE recycling endosomal tubules

**Fig 5:**
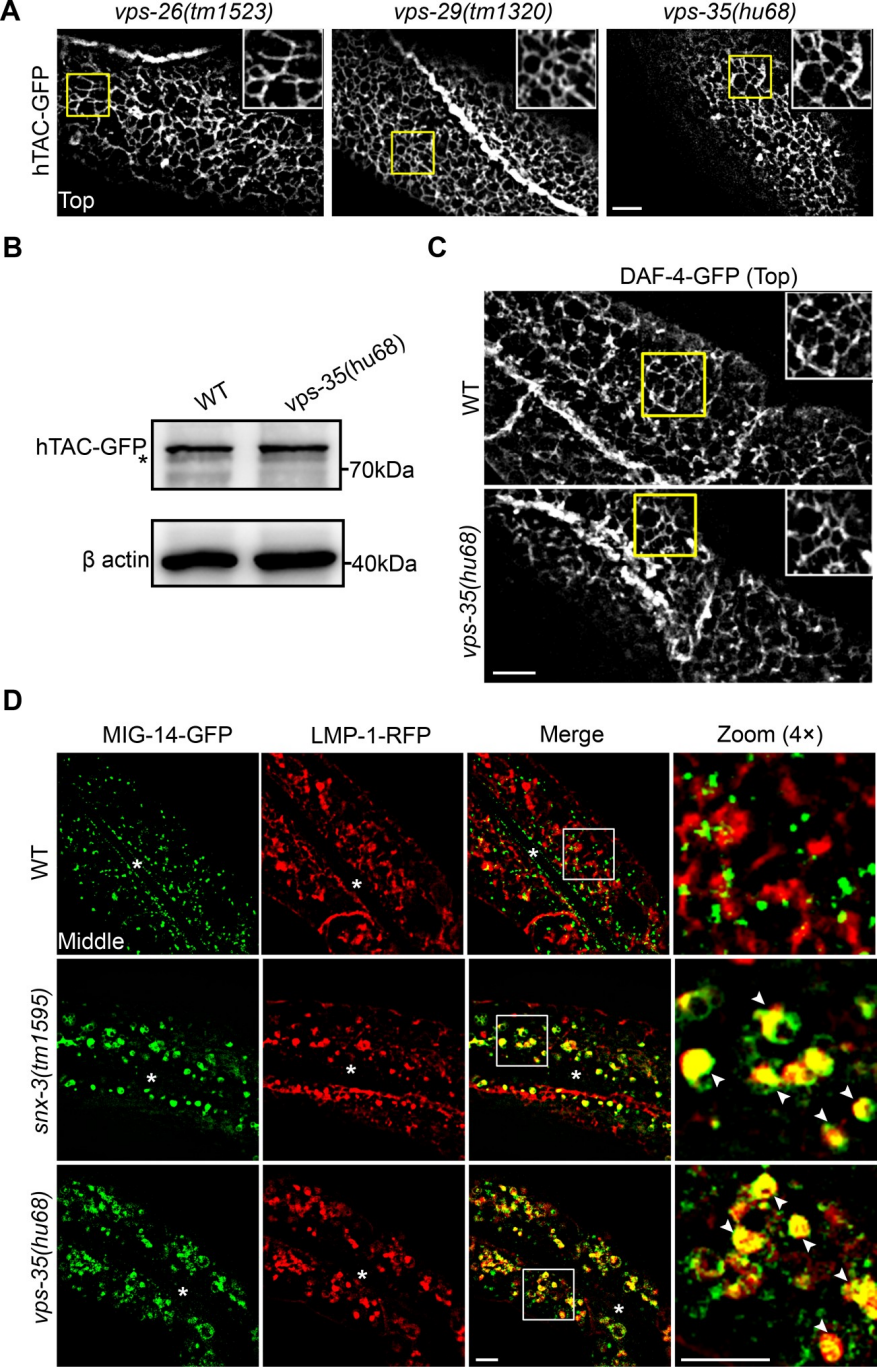
The retromer trimeric complex is dispensable for the hTAC-containing recycling endosomal tubules. (A) Micrographs showing the tubular profiles of subplasmalemmal hTAC-GFP-containing carriers in each of the *vps-26/-29/-35* deletion mutants. (B) Western blot showing the protein level of hTAC-GFP in the *vps-35(hu68)* mutants is comparable to that in WT animals. The asterisk depicts non-specific band. (C) Micrographs showing the tubular profile of subplasmalemmal DAF-4-GFP-containing carriers in the *vps-35(hu68)* mutants. (D) Confocal images showing colocalization between MIG-14-GFP and lysosomes labeled by LMP-1-RFP. Compared with the WT animals, the overlap level of MIG-14-GFP with LMP-1-RFP is significantly increased in *snx-3(tm1595)* mutants and *vps-35(hu68)* mutants, respectively. The arrowheads indicate positive overlap. Asterisks depict the intestine lumen. Scale bars: 5 μm.

The retromer comprising VPS26/29/35 is the core sorting complex at the SEs. To determine if SNX-3 coordinates with the retromer complex to mediate the recycling of the CIE cargoes, the distribution of basolateral subplasmalemmal hTAC-GFP was examined for the deletion alleles *vps-26(tm1523)*, *vps-29(tm1320)* and *vps-35(hu68)* [23], respectively. Strikingly, the hTAC-GFP-loaded tubular endosomes were unaltered in the three mutant animals (Fig 5A). Quantification of the Western blot analysis showed that the steady-state protein level of hTAC-GFP in mutants lacking the core retromer subunit VPS-35 was comparable to that in WT animals (Fig 5B). Similarly, the DAF-4-GFP tubular endosomes were preserved in *vps-35(hu68)* mutants or upon RNAi treatment against the retromer components (Figs 5C and S6). In all, these results strongly suggested that the retromer trimer is dispensable for the recycling of these ARF-6-associated CIE cargoes. MIG-14, the *C*. *elegans* homolog of the Wnt-secretion factor Wntless, has been identified as a clathrin-dependent retrograde cargo and is retrieved by the SNX3-retromer complex at EEs and delivered to the TGN [14,41,42]. We then examined whether the SNX-3-retromer complex was involved in the post-endocytic trafficking of the intestinal integrated MIG-14. The punctate MIG-14-GFP signal in the WT cytosol did not colocalize with LMP-1-RFP, a marker of the late endosome (LE) and lysosomes. However, in both *snx-3(tm1595)* and *vps-35(hu68)* mutant animals, MIG-14-GFP overlapped with LMP-1-RFP prominently (Fig 5D). Together, these data support that, different from the retrograde transport of Wntless from EEs to TGN, which is mediated by SNX3-retromer complex, the recycling of the ARF-6-associated cargoes, hTAC and DAF-4, to the PM does not require the retromer trimeric complex.

To further evidence that SNX-3 mediates CIE recycling independently from the retromer, we examined the colocalization of SNX-3 or retromer components with the hTAC-containing tubular endosomes. We constructed a functional *vha-6*-driven RFP-VPS-35 plasmid, which rescued the abnormal accumulation of MIG-14-GFP when injected in *vps-35(hu68)* mutants (S7A Fig). We found that a significant fraction of SNX-3 (57.4%) decorated the tips of hTAC-containing tubular-vesicular structures, whereas very little amount of VPS-35 located in the vicinity or at the hTAC-containing structures (15%) (S7B–S7E Fig). In addition, decoration of RFP-SNX-3 on the hTAC-GFP tubules was not affected in the *vps-35(hu68)* mutant (S7B and S7C Fig). Moreover, only a partial colocalization (43.6%) was observed between GFP-SNX-3 and RFP-VPS-35 (S7F and S7G Fig). These results demonstrated that SNX-3 might function independently of the retromer complex.

### SNX-3-mediated CIE recycling tubules do not converge into the classical EEA-1-labeled sorting endosomes

**Fig 6:**
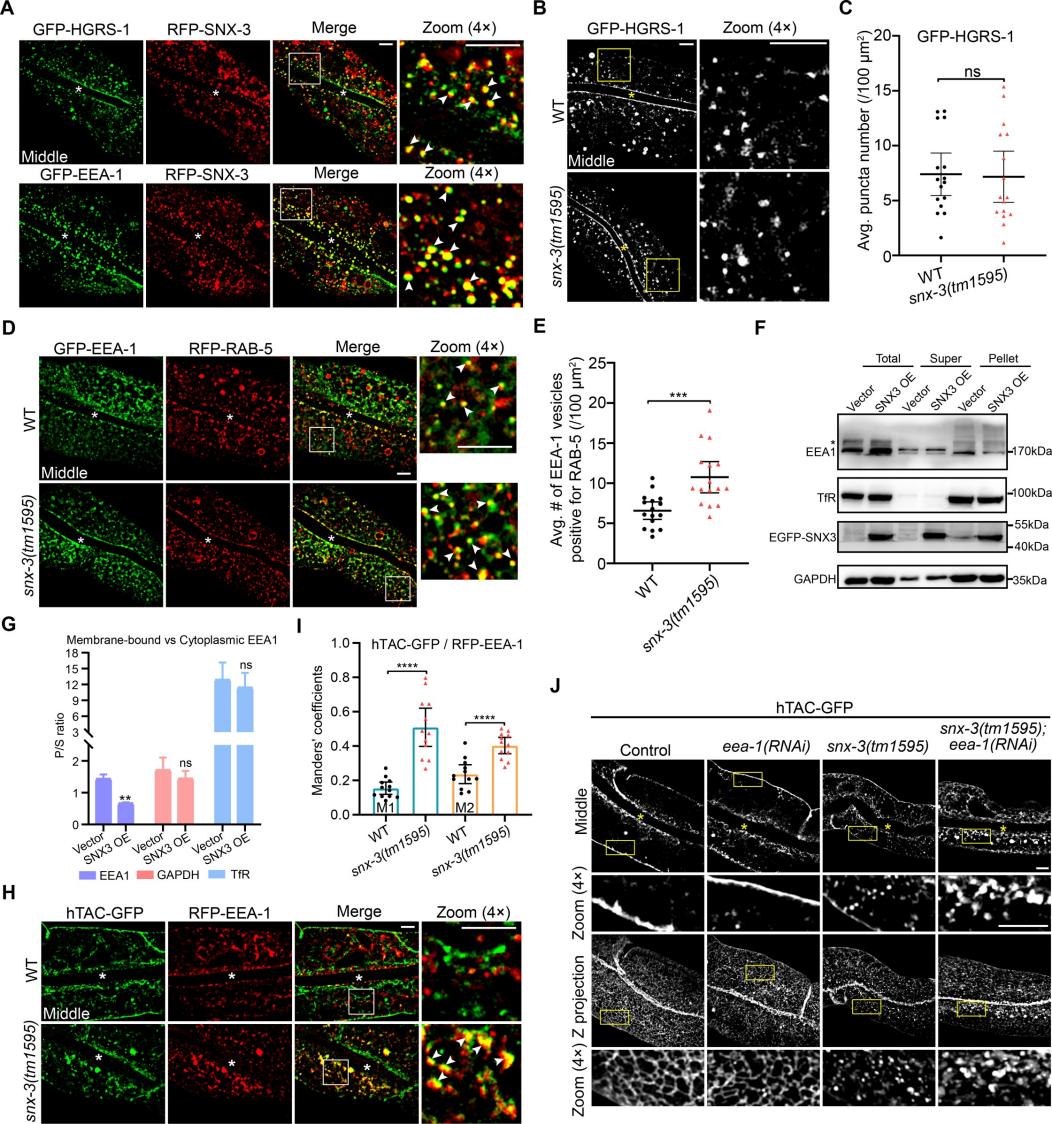
SNX-3-mediated CIE recycling tubules do not converge into the classical EEA-1-labeled sorting endosomes. (A) Micrographs showing RFP-SNX-3 partially overlapped with GFP-tagged HGRS-1 or EEA-1. (B,C) Confocal images (B) and quantification of the number of GFP-HGRS-1 signals (C) show no impact on GFP-HGRS-1 in *snx-3(tm1595)* mutants. [ROI] = 16, n = 12. Error bars are mean ± 95% CI. ns, not significant (Student’s *t* test). (D) Confocal images showing the overlap of GFP-EEA-1 and RFP-RAB-5 in WT and *snx-3(tm1595)* mutant animals. (E) Quantification of the number of RFP-RAB-5-positive GFP-EEA-1 vesicles for each genotypes as indicated in D. Error bars are mean ± 95% CI ([ROI] = 16, n = 12). ***P<0.001 (*t* test with Welch’s correction). (F,G) The membrane-to-cytosol ratio of EEA1 decreased in SNX3-overexpressed Hela cells. Hela cells overexpressing empty or EGFP-SNX3 vector were separated into a supernatant (S) fraction and a pellet (P) fraction and were stained for endogenous EEA1, TfR (as a control of transmembrane protein), GAPDH, and ectopically expressed EGFP-SNX3 (F). The asterisk depicts non-specific band. The amount of individual proteins in the supernatant and pellet fractions was quantified using densitometry and is shown as a P/S ratio (G). Data are presented as mean ± SEM and represent three independent experiments. ns, not significant; **P<0.01 (Student’s *t* test). (H,I) Confocal images showing the overlap of RFP-EEA-1 and hTAC-GFP significantly increased in *snx-3(tm1595)* mutants (H) and the Manders’ coefficients were calculated (I). Error bars are mean ± 95% CI (WT: [ROI] = 12, n = 9; *snx-3(tm1595*): [ROI] = 12, n = 7). ****P<0.0001 (*t* test with Welch’s correction for M1; Student’s *t* test for M2). M1: green pixels overlapping red; M2: red pixels overlapping green. (J) Confocal images showing the subcellular distribution of intestinal hTAC-GFP. The middle section and Z-projection images showed RNAi treatment of *eea-1* did not disrupt the subplasmalemmal tubular profiles of hTAC-GFP; concomitant depletion of *snx-3* and *eea-1*, however, led to obvious accumulation of clustered hTAC-GFP signals in the cytosol. In A, B, D, H, and J, the arrowheads indicate positive overlap. Asterisks depict the intestine lumen. Scale bars: 5 μm. Quantitative data are available in S1 File.

**Fig 7:**
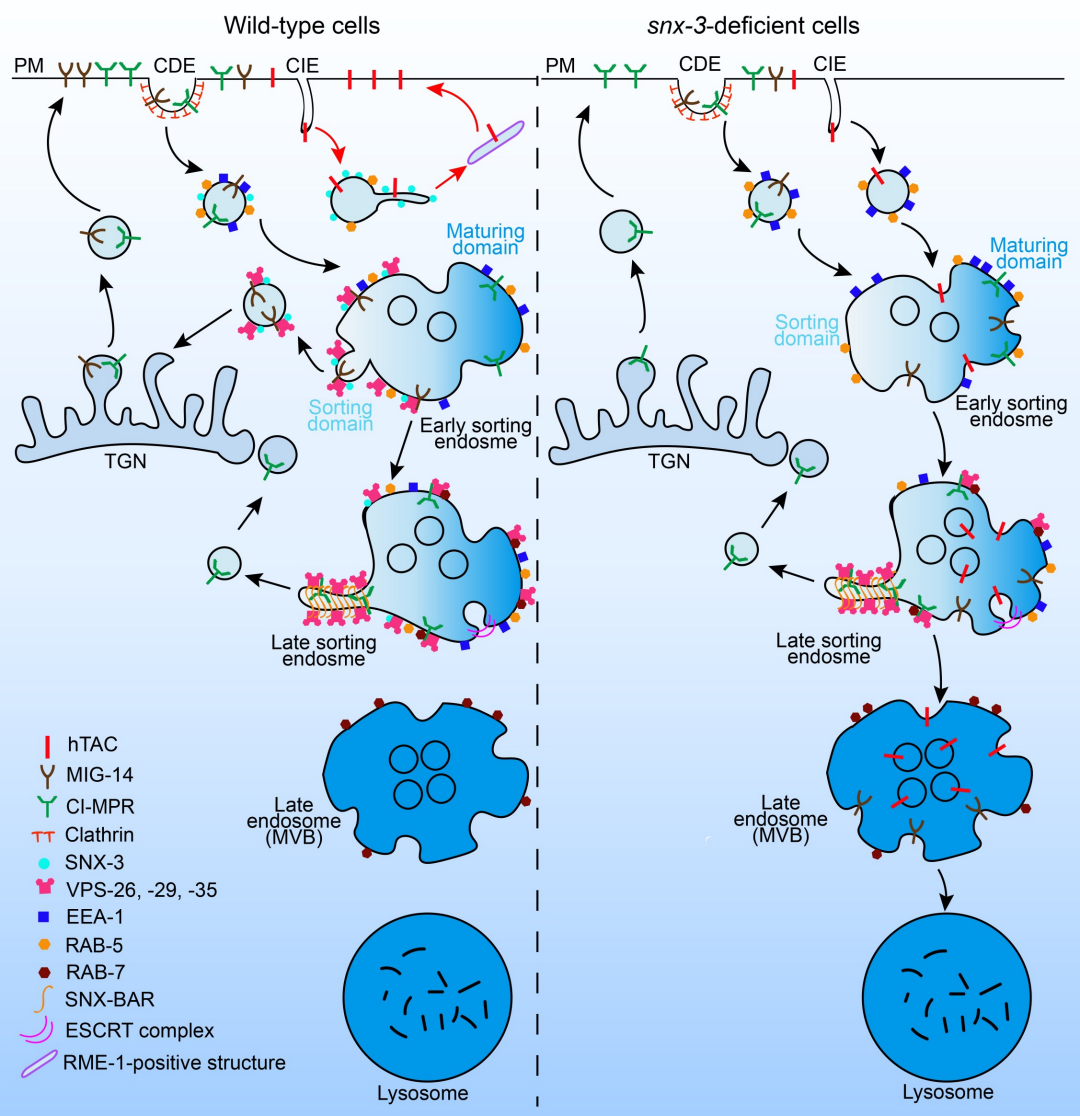
Schematic of SNX-3-promoted tubule-vesicular exit across the endosomal maturation pathway. In wild-type cells, SNX-3 promotes tubular endosomal recycling of specific CIE cargoes from EEA-1-negative EEs, which is independent of retromer; SNX-3-retromer complex subsequently forms in SEs and facilitates retrograde transport of several CDE cargoes. When SNX-3 is suppressed, EEA-1 is increasingly recruited to the CIE-derived subpopulation of EEs, then facilitates fusion of these endosomes with classical SEs and consequently leads to mis-trafficking of CIE cargoes (e.g. hTAC) into lysosomes via ESCRT pathway.

How SNX-3 mediates the trafficking of hTAC to the PM independently from the retromer trimer remains to be determined. Using an *in vitro* lipid-binding assay, Chua *et al* previously showed that SNX3 and EEA1 compete with each other for binding to PtdIns(3)P lipids [43]. It prompted us to test whether the loss of SNX-3 led to spatiotemporal imbalance of the PtdIns(3)P-binding endosomal trafficking regulators on the EEs or SEs. EEA1 and Hrs are two major FYVE domain-containing PtdIns(3)P-binding trafficking regulators [44]. GFP-tagged HGRS-1 and EEA-1 both displayed a partial overlap with RFP-SNX-3 in WT intestines (Fig 6A), suggesting a spatiotemporal difference in their recruitment to the dynamic EEs/SEs. The loss of SNX-3 did not prominently change the number of GFP-HGRS-1 puncta (Fig 6B and 6C), as observed by Norris *et al* in the coelomocyte of *C*. *elegans* [37]. However, the amount of EEs-located GFP-EEA-1 was increased by about 63% (Fig 6D and 6E). These data indicated that the loss of SNX-3 did not increase the amount of degradative ESCRT microdomains but augmented the quantity of EEA-1-positive subdomains on EEs. To further confirm the competition between SNX3 and EEA1 for binding to EEs, we separated the cytosol from the membrane structures by ultracentrifugation of lysates of mock and EGFP-SNX3 overexpressed HeLa cells. We analyzed the amount of EEA1 in each fraction by western blotting (Fig 6F). The membrane-to-cytosol ratio of EEA1 dropped by 53% in cells overexpressing EGFP-SNX3 (Fig 6G), supporting a competitive relationship between SNX3 and EEA1 for the association with PtdIns(3)P-enriched EEs. Moreover, in agreement with a previous report [45], little EEA-1 decorated the hTAC-GFP membranous tubules in the WT intestines. However, the overlap of hTAC-GFP puncta with EEA-1 was prominently increased in the *snx-3(tm1595)* mutant animals [Manders’ coefficient M1 = 0.15 vs. 0.51, M2 = 0.24 vs. 0.40, for WT vs. *snx-3(tm1595)* mutant animals] (Fig 6H and 6I). As known, EEA1 plays an important role in tethering incoming endocytic vesicles with the EEs and is potentially important for the dynamic early endosomal membrane fusion and remodeling [46]. Our results indicated that the internalized hTAC-GFP enter the SNX-3-mediated CIE recycling tubules for its efficient recycling to the PM which does not converge into the classical EEA-1-labeled SEs. The loss of SNX-3 probably leads to an increased recruitment of EEA-1 to the fragmented hTAC-laden CIE endosomal intermediates, resulting in the capture of hTAC by the ESCRT complex (Fig 7).

To verify this hypothesis, we further compared the effect of loss of SNX-3, EEA-1 or both on the steady-state distribution of hTAC. The RNAi treatment against *eea-1* had no obvious effect on the subcellular distribution of hTAC, whereas *snx-3(tm1595); eea-1(RNAi)* double mutants resulted in severe accumulation of hTAC puncta in the cytosol and a diminished basal membrane-associated signal (Fig 6J), which may be due to blockage of transport toward the recycling and degradative pathways.

## Discussion

The steady-state localization of transmembrane proteins in the endocytic system is the consequence of many sorting events that occur at various points throughout the endosomal pathway. It remains controversial as to how many independent types of recycling transport carriers are formed from endosomes and what their relative relationship is. Previous studies showed that SNX3, in concert with the retromer trimer complex, is required for retrograde recycling of Wntless from EEs to TGN. Here we identified SNX-3 as a novel regulator, independent of the retromer trimer, mediating tubular endosomal recycling of CIE cargoes in the *C*. *elegans* intestine (Fig 7).

Several lines of evidence support a function of SNX-3 independently of the retromer complex. First, the endosomal localizations of SNX-3 and VPS-35 only partially overlap, suggesting the existence of two pools of SNX-3, one is retromer complex-positive, and the other one is negative. Hong’s lab previously revealed that SNX3 is enriched in a subdomain of the EE and, to a smaller extent, in the recycling endosome [27]. The retromer trimer, however, is mainly restricted to maturing sorting endosomes, partially because its membrane recruitment needs the cooperation of RAB7 GTPase and SNX3 [47,48]. Accumulating evidence also suggests that SNX proteins, in addition to binding to membranes, play a critical role in the cargo selection. One intriguing model proposed that the membrane tubulating BAR domain-containing SNX5/6 interacts with a sorting signal shared by over 60 cargoes, whereas SNX1/2 interacts with PtdIns(3)P to trigger endosomal recruitment [11]. In the present study, we found that SNX-3 but not VPS-35 locates to the tips of hTAC-containing tubules, which indicates that SNX-3 may function independently of the retromer trimer.

Our work shows that the tubule-vesicular exit promoted by SNX-3 across the endosomal maturation pathway displays distinct cargo enrichment. The exit on subdomains of the EEA-1-negative EEs prefers the ARF-6-associated CIE cargo hTAC to CDE cargo MIG-14 and promotes transport toward the cell surface recycling pathway. By contrast, the exit on the EEA-1-positive SEs prefers MIG-14 to hTAC and promotes transport toward the retrograde pathway (Fig 7). Our results are consistent with a model in which the spatial separation of SNX-3 and the SNX-3-retromer along the endosomal maturation pathway contributes to achieving a differential sorting. Our investigation also evidenced the competition between SNX-3 and EEA-1 for binding to PtdIns(3)P-enriched EEs and its influence on the subdomains of EEs to proceed to direct recycling or to maturing SEs (Fig 7). The loss of SNX-3 led to the capture of hTAC or DAF-4 into the MVBs via the ESCRT pathway for further lysosomal degradation. Although we did not provide evidence on the ubiquitination state of the lysosome-targeted hTAC, Mark von Zastrow and his colleagues previously found that components of the ESCRT complex, Hrs and VPS4, are involved in the lysosomal trafficking of non-ubiquitinated delta opioid GPCR in mammalian cells [49]. This suggests that the ESCRT pathway may mediate post-endocytic transport to lysosomes of ubiquitinated and non-ubiquitinated cargo proteins. Endosomes are highly dynamic organelles that comprise distinct subdomains occupied by different RAB proteins and PtdInsPs. The presence of RAB5, PtdIns(3)P, and EEA1 is defined as the “classical” SEs [3]. Interestingly, our previous work revealed that decomposition of the exocyst complex caused fragmentation of the hTAC-containing recycling tubules and an increase of RAB-10 localization on these fragmented transport carriers [21]. Here, we found that the loss of SNX-3 led to fragmentation of hTAC-containing tubules and misrouting of hTAC into lysosomes for degradation, which however was blocked by EEA-1 depletion. Our results support the idea that EEA1-positive endosomes might be a selection point for entry into the MVB pathway and lysosomal degradation [50]. Coincidentally, overexpression of SNX3 impairs transport from EEs toward LEs, most likely by inducing the accumulation of multivesicular regions on early endosomal membranes [51]. Therefore, we propose that SNX3, which presents the simplest structure among the SNX family members, perhaps possesses the versatile abilities to regulate post-endocytic transport along the endo-lysosomal pathway.

Our present work does not answer how such tubular membrane carriers are formed in the SNX-3-mediated CIE recycling pathway. Unlike the SNX-BAR proteins which induce membrane tubulation via their BAR domains, the molecular mechanisms by which PX domain-only SNX3 mediates the membrane remodeling and the formation of endosomal transport carriers remains unclear. Our data does not support the possibility that SNX3 interacts with SNX1/2-SNX5/6 for membrane remodeling as SNX27 does [1]. Indeed, Harterink *et al* demonstrated that SNX-BAR components and SNX3 were not co-immunoprecipitated, suggesting that SNX3 and SNX-BAR do not coexist in the same complex [14]. Moreover, our imaging-based screening experiments showed that depletion of the SNX-BAR components does not affect the tubular aspect of hTAC-containing carriers. However, our initial screening results suggested that the loss of SNX-27 partially disrupt the hTAC-containing tubules. SNX27 is a PDZ (PSD95, Dlg, ZO1) domain-containing SNX protein that usually interacts with cargo proteins harboring the PDZ-ligand [52]. However, hTAC and DAF-4 do not harbor the PDZ-ligand. It is necessary for us to further investigate whether SNX-3 and SNX-27 work together or sequentially along the endosomal maturation pathway for recycling of such CIE cargoes. Moreover, Donaldson’s lab previously identified Hook1, a microtubule- and cargo-tethering protein, sorts a set of clathrin-independent cargoes (including CD44 and CD98) into recycling tubules [53]. Depletion of Hook1 altered the trafficking of these CIE cargoes toward EEA1 compartments. However, depletion of ZYG-12, the ortholog of human Hook1 in *C*. *elegans*, has no any obvious impact on the tubular profile of hTAC-containing endosomes (S8 Fig), indicating that SNX3 and Hook1 mediate independent recycling tubular carriers. Further work needs to understand how SNX3 cooperates with other proteins for mediating the sorting and transport through the endosomes.

## Materials and methods

### General methods and strains

All *C*. *elegans* strains used in this study were derived originally from the wild-type Bristol strain N2. Worm cultures, genetic crosses, and other strain manipulation methods were essentially those described by Brenner [54]. Worms were grown on OP50 *E*. *coli*-seeded nematode growth medium (NGM) plates at 20°C. A complete list of mutant and transgenic strains used in this study can be found in S1 Table. RNAi-mediated interference was performed as described previously [55]. All bacterial RNAi feeding constructs were derived from the Ahringer library, controls were treated with bacterial strain HT115(DE3), which was transformed with empty L4440 feeding vector [56]. For most RNAi experiments, synchronized L1 stage animals were cultured for 60 h and scored as young adults.

### Plasmids and generation of transgenes

The transcriptional reporter constructs, *Psnx-3*::GFP and *Psnx-27*::GFP, were generated as previously described [21,57]. Briefly, the 690-bp promoter of *snx-3* and 4.978-kb promoter of *snx-27* were amplified by PCR and subcloned into the vector pPD95.75 (a gift from the Fire lab) at the SalI and KpnI sites, respectively. The full length *snx-3* cDNA was digested with Xmal and KpnI, and cloned into the *Pvha-6*::*TagRFP-T* vector to generate *Pvha-6*::*TagRFP-T*::*SNX-3*. The N-terminal and C-terminal deletion mutants for *Pvha-6*::*TagRFP-T*::*SNX-3* were generated by PCR overlap extension method [58], and *Pvha-6*::*TagRFP-T*::*SNX-3(Y71A)* was produced by PCR-based missense mutagenesis. To construct *Pvha-6*::*TagRFP-T*::*VPS-35*, *Pvha-6*::*TagRFP-T*::*EEA-1* and *Pvha-6*::*TagRFP-T*::*HGRS-1*, the genomic DNA of *vps-35* or *eea-1* and the cDNA of *hgrs-1* were amplified and individually inserted into the vector *Pvha-6*::*TagRFP-T*, through Xmal and KpnI sites. The *lmp-1* cDNA fused by TagRFP-T with a linker sequence Gly-Ala at 3′-terminus was amplified and cloned into *Pvha-6*::*TagRFP-T* by using Xmal and KpnI sites to generate *Pvha-6*::*LMP-1*::*TagRFP-T*. *Pvha-6*::*GFP*::*EEA-1* and *Pvha-6*::*GFP*::*SNX-3* were constructed by ligating *eea-1* genomic DNA or *snx-3* cDNA into *Pvha-6*::*GFP* vector at NheI and KpnI sites, respectively. The *Pvha-6*::*TagRFP-T*::*RAB-5* were generated previously [28]. To generate *Pvha-6*::*ARF-6*::*mCherry*, the *arf-6* cDNA was amplified and inserted into *Pvha-6*::*mCherry* vector at the Smal and Agel sites. To construct pEGFP-SNX3 for expression in Hela cells, the full length cDNA of mammalian *SNX3* was amplified from the plasmid GST-SNX3 (gift from Prof. Wanjin Hong, Institute of Molecular and Cell Biology, Singapore) and inserted into pEGFP-C1 vector at BglII and EcoRI sites.

The *Pvha-6*::*TagRFP-T*::*SNX-3* MosSCI line was generated by the direct insertion method as previously described [21,26]. The *Pvha-6*::*TagRFP-T*::*SNX-3* sequence was cloned into an AflII-SbfI digested MosSCI vector pCFJ151 (gift from Prof. E. M. Jorgensen, University of Utah, Salt Lake City) to produce repair template. The injection mix of 50 ng/μl repair template and 50 ng/μl Mos1 transposase pJL44 (*Phsp-16-48*::*transposase*) was coinjected into EG4322 (*ttTi5605; unc-119(ed3)*) animals. Injected worms were transferred to standard NGM plates (5–6 worms per plate) and placed at 20°C. 72 h post-injection, fifty individual *unc-119*-rescued F1 worms with wild-type movement were picked out, transferred to standard NGM plates and further allowed to exhaust the bacterial food source. Two weeks later, plates containing transgenic lines were screened for insertion events based on the *unc-119-*rescued phenotype and intestinal fluorescence of *Pvha-6*::*TagRFP-T*::*SNX-3* transgene. Six lines were picked out and verified for true insertion at target site by using PCR analysis. The primers used were 5’-TCTGGCTCTGCTTCTTCGTT-3’ and 5’-CAATTCATCCCGGTTTCTGT-3’. One line without aggregated RFP-SNX-3 fluorescent signals was finally chosen by using fluorescence microscopy for further analysis in this study.

### Western blot and quantitative real-time PCR gene expression analysis

Different genotypic worms were synchronized and cultured on NGM plates. Animals were washed off from three 60-mm-diameter plates with M9 buffer (22 mM KH_2_PO_4_, 22 mM Na_2_HPO_4_, 85 mM NaCl, 1 mM MgSO_4_) and collected into a 1.5 ml eppendorf tube, centrifuged 1 min at 3,000 rpm. The worm pellet was resuspended with RIPA lysis buffer (50 mM Tris pH 7.4, 150 mM NaCl, 1% Triton X-100, 1% sodium deoxycholate and 0.1% SDS) supplemented with 1 mM PMSF on ice and crushed by squeezing and rotating the grinding rod, centrifuged at 12,000 rpm for 30 min at 4°C. The supernatant were collected and protein concentration was measured by using BCA Protein Assay kit (Beyotime, P0010S). Equal protein amounts were mixed with loading buffer (LSB), boiled, and subjected to 10% SDS-PAGE followed by transferring to nitrocellulose membranes (Millipore, Bedford, MA) for immunoblotting. Prior to antibody incubation, Membranes were blocked in blocking buffer (5% nonfat dry milk in TBS and 0.1% Tween-20) for 2 h at room temperature. Mouse monoclonal anti-β actin (proteintech 66009-1-Ig, 1:20,000) and Mouse monoclonal anti-GFP (Santa Cruz sc-9996, 1:500) antibodies were incubated overnight at 4°C. Membranes were washed three times in 0.1% Tween-20/TBS, followed by incubation with an anti-mouse IgG horseradish peroxidase-conjugated secondary antibody (1,20,000) for 2 h at room temperature. Proteins detection was performed by using SuperSignal West Femto Maximum Sensitivity Substrate (Thermo Fisher Scientific, Waltham, MA, USA). Semi-quantitative analysis of the bands was carried out using ImageJ. β actin was used as an internal reference. The mean grey value of hTAC-GFP band to internal reference band was considered as the relative protein expression. Three parallel wells were set for each protein sample.

The procedure of qRT-PCR analysis of *hTAC* and *snx-3* gene expression was performed as previously described [28]. The primers used were 5’-GATTCATACCTGCTGATGTG-3’ and 5’-ACATGGTTCCTTCCTTGTAG-3’ for *hTAC*, 5’-AGAAAGAATCAAGCGTGCGT-3’ and 5’-AATTCCATCATCGGATCGGA-3’ for *snx-3*, 5’-TGCTTGCTGGGAGCTCTACTGTCTC-3’ and 5’-CAACAACAGTTGGCTCAAGATCTAC-3’ for *tba-1*.

### Membrane fractionation assay

Hela cells expressing control vector or EGFP-SNX3 were grown on 10-cm-diameter culture plates for 24h, washed with ice-cold PBS and harvested in 500 μl of ice-cold homogenization buffer (20 mM HEPES, PH 7.4, 250 mM sucrose and protein inhibitors). The cells were lysed with 10 passes through a 25-gauge needle. After centrifugation at 1000 × *g* for 10 min at 4°C to remove unbroken cells and cellular debris, the supernatant was ultracentrifugated at 60,000 rpm for 1 h at 4°C in a Beckmann TLA-110 Rotor to yield a pellet of total cellular membranes and a supernatant representing the cytosolic fraction. The pellets were resuspended in SDS lysis buffer (50 mM Tris,1% SDS, 50 mM DTT and protein inhibitors). Equal fractions of cytosol and membrane were analyzed by SDS-PAGE followed by immunoblotting using mouse monoclonal anti-GAPDH (Proteintech 60004-1-Ig, 1:5000), mouse monoclonal anti-GFP (Proteintech 660021-1-Ig, 1:1000), mouse monoclonal anti-CD71 (Proteintech 66180-1-Ig, 1:1000), and mouse monoclonal anti-EEA1 (BD 610457, 1:1000) antibodies. Quantitation of Western blots was performed using ImageJ.

### Microinjection into the pseudocoelom

To disrupt the function of lysosomes or proteasomes in the intestine of *C*. *elegans*, 500 nM BafA1 (Selleck, S1413) or 50 μM MG132 (Selleck, S2619) was injected into the pseudocoelom of young adult hermaphrodites 6 h before imaging. The drugs were diluted in DMSO and used at a final concentration of 0.5% DMSO in egg buffer (118 mM NaCl, 48 mM KCl, 2 mM MgCl_2_, 2 mM CaCl_2_, and 25 mM Hepes pH7.3). The treated worms were used for confocal imaging.

### Confocal microscopy

Live young adult worms were mounted on a 2% (wt/vol) agarose pad with 10 mM levamisole (Sigma-Aldrich). Fluorescence images were obtained using a spinning-disk confocal system (CSU-X1 Nipkow; Yokogawa) equipped with an EM CCD camera (DU897K; ANDOR iXon) and oil-immersion objectives (60× N.A. 1.45, 100× N.A. 1.3 or 150× N.A. 1.45). Two 50 mW solid state lasers (491 nm and 561 nm) coupled to an acoustic-optical tunable filter (AOTF) were used to excite GFP and RFP, respectively. The method to reduce interference from autofluorescence was previously described [21]. Z-series of optical sections were acquired using 0.3 μm step size.

### Imaging analysis

Images were collected by using Andor IQ 3.3 software and analyzed in ImageJ 1.52f (Wayne Rasband, National Institutes of Health). Z-stack images were 3D deconvolved by using AutoQuant X2 (Media Cybernetics) software. In most cases, single top or middle section of images was displayed; for images with low signal-to-noise ratio, two to three sections were processed to yield maximum intensity projections and displayed. The brightness and contrast of images were adjusted and a median filter or a background subtraction method was used to decrease noise. Fluorescence intensities were quantified with ImageJ. The tubule length was calculated by using ImageJ plugin “tubulength”. For quantification of the number or the occupied areas of endosome structures, images were preprocessed with 1.0-pix-wide median filter and 10-pix-wide rolling-ball background subtraction to increase the signal-to-noise ratio. Then the region of interest (ROI) (100 pix * 100 pix) was cropped and the maximum threshold value was automatically set by imageJ. The minimum threshold value was manually adjusted by moving the “Upper slider” of the “Threshold” tool and a manual inspection is needed to ensure the outlines of the low-fluorescent objects are separated from the background. Finally, ImageJ plugin “Analyze Particles” was used to measure the features of endosomal structures.

### Colocalization analysis

Pearson’s or Manders’ coefficient was used for most cases by using ImageJ plugin “JACop”. Colocalization percentage was scored as the ratio of the green/red puncta signals that are positive for red/green signals to the total number of green/red puncta.

### Statistical analysis

Statistical significance was calculated with Graphpad Prism 8 (GraphPad Software, La Jolla, CA). All data were tested for normality using Anderson-Darling test. An unpaired two-tailed Student’s *t* test was used to determine significant differences between two groups and Welch’s correction was performed for unequal variance. One-way ANOVA followed by a Tukey’s *post hoc* test was used to evaluate differences between three or more groups if samples were normally distributed and had equal variance. Brown-Forsythe and Welch ANOVA with Dunnett T3 multiple comparison test was conducted if samples had non equal variance. If the dataset was not normally distributed, Kruskal-Wallis test with Dunn’s *post hoc* test for multiple comparison was conducted. No inclusion or exclusion criteria were applied during analysis. The worms were selected randomly within the same genotype. The Histogram and cumulative distributions of RFP-RME-1 structures’ area were plotted by Origin 2018 (OriginLab).

## Supporting information

S1 Fig (A) Micrographs showing the subplasmalemmal hTAC-GFP or DAF-4-GFP tubules overlapped with ARF-6-mCherry-positive structures. The arrowheads indicate overlap. (B) Confocal images showing the steady-state distributions of two clathrin-dependent cargo proteins, the intestine-integrated hTfR-GFP and oocyte-internalized YP170-GFP, are comparable in *snx-3(tm1595)* mutants to those in WT animals. Oocytes proximal to the spermatheca are numbered as -1. Scale bars: A, 5 μm; B, 20 μm.
(TIF)

S2 Fig The expression profiles of SNX-3 and SNX-27 in *C*. *elegans*. Confocal images of adult hermaphrodites expressing GFP driven by the *snx-3* promoter (690 bp upstream of the start codon) (A) or the *snx-27* promoter (about 5 kb upstream of the start codon) (B). GFP is broadly expressed in *C*. *elegans*, including head and tail neurons, intestine, hypodermis, muscle, vulva, reproductive system and coelomocytes. Scale bars: 20 μm.
(TIF)

S3 Fig Single-copy insertion of *Pvha-6*::*RFP*::*SNX-3*. (A) Schematic of the targeting construct containing the *unc-119(+)* rescue gene and *Pvha-6*::*RFP*::*SNX-3* transgene flanked by DNA homologous to the *ttTi5605mos* insertion site. (B) PCR verification of inserted *Pvha-6*::*RFP*::*SNX-3* transgenes. The forward primer anneals to the genomic region outside of that contained in the targeting construct, and the reverse primer is in the *unc-119(+)* selectable marker. A PCR band of expected size (1.7 kb) was amplified only from the insertion strain. (C) PCR analysis of *RFP*::*SNX-3* mRNA expression levels shows the mRNA level is lower in *Pvha-6*::*RFP*::*SNX-3* transgene insertion strain than in *Pvha-6*::*RFP*::*SNX-3* extragenic strain. The forward primer anneals to the C-terminal region of *RFP*, and the reverse primer anneals to the N-terminal region of *SNX-3*.
(TIF)

S4 Fig Effect of the different regions of SNX-3 on the hTAC-containing endosomal tubules. (A) Schematic diagram illustrating the domain architecture of SNX-3. (B) Confocal images show the fragment of N-terminus-deleted SNX-3 restore the tubular morphology of hTAC-containing structures in *snx-3(tm1595)* mutants, while the single point-mutated SNX-3(Y71A) or the fragment of C-terminus-deleted SNX-3 loses the capability. The Arrows indicate basolateral PM-associated hTAC-GFP. Asterisks depict the intestine lumen. Scale bar: 10 μm.
(TIF)

S5 Fig DAF-4 is mediated by ESCRT pathway into the lysosome for degradation in *snx-3(tm1595)* mutants. (A) Micrographs showing the colocalization of DAF-4-GFP with LMP-1-RFP in *snx-3(tm1595)* mutants pretreated with BafA1. In WT animals, no obvious colocalization of DAF-4-GFP and LMP-1-RFP signals is observed either in the absence (7.5%) or presence (7.8%) of BafA1. By contrast, BafA1 treatment leads to accumulation of DAF-4-GFP that prominently overlapped with LMP-1-RFP signals in *snx-3(tm1595)* mutants (47.1%), indicating DAF-4 is efficiently degraded in the lysosome of *snx-3(tm1595)* mutants. (B) Colocalization of DAF-4-GFP with LMP-1-RFP was calculated for each condition as depicted in A. [ROI] = 21/18/19/19, n = 13/10/14/10 for WT(-)/*snx-3(tm1595)*(-)/WT(+)/*snx-3(tm1595)*(+). Error bars are mean ± 95% CI. ns, not significant; ****P<0.0001(Brown-Forsythe and Welch ANOVA with Dunnett T3 multiple comparison test). (C) Confocal images showing the overlap of DAF-4-GFP with RFP-HGRS-1 is increased in *snx-3(tm1595)* mutants. (D) Manders’ coefficients for DAF-4-GFP and RFP-HGRS-1 as depicted in C were calculated, M1 = 0.12 vs. 0.30, M2 = 0.14 vs. 0.36, for WT vs. *snx-3(tm1595)* mutant animals, error bars are mean ± 95% CI (WT: [ROI] = 17, n = 9; *snx-3(tm1595)*: [ROI] = 16, n = 10). ****P<0.0001 (*t* test with Welch’s correction for M1; Student’s *t* test for M2). M1: green pixels overlapping red; M2: red pixels overlapping green. In A and C, asterisks depict the intestine lumen, arrowheads indicate positive overlap. Scale bars: 5 μm. Quantitative data are available in S1 File.
(TIF)

S6 Fig The retromer trimer is not involved in the endosomal recycling of DAF-4. The tubular profile of subplasmalemmal DAF-4-GFP-containing carriers is not changed upon RNAi treatment of *vps-35*, *vps-26*, *or vps-29*. Scale bars: 5 μm.
(TIF)

S7 Fig VPS-35 is not involved in the formation of the hTAC-containing endosomal tubules. (A) Confocal images show the transgene of *vha-6* driven RFP-VPS-35 restore the punctate morphology of MIG-14-GFP in *vps-35(hu68)* mutant animals, indicating the RFP-tagged VPS-35 is functional. (B) Micrographs showing the localization of RFP-SNX-3 at the tips of hTAC-GFP tubules in WT and *vps-35(hu68)* mutant animals. (C) Percentage of RFP-SNX-3 overlapped with hTAC-GFP as depicted in B was calculated, error bars are mean ± 95% CI (WT: [ROI] = 26, n = 9; *vps-35(hu68)*: [ROI] = 23, n = 9). ns, not significant (Student’s *t* test). (D) Micrographs showing very few RFP-VPS-35 locate at hTAC-containing structures. (E) Percentage of RFP-VPS-35 overlapped with hTAC-GFP as depicted in D was calculated, error bars are mean ± 95% CI ([ROI] = 17, n = 4). (F) Micrographs showing partial overlap of co-expressed GFP-SNX-3 and RFP-VPS-35 in the *C*. *elegans* intestine. (G) Percentage of GFP-SNX-3 overlapped with RFP-VPS-35 as depicted in F was calculated, error bars are mean ± 95% CI ([ROI] = 27, n = 21). In A, B, D, and F, asterisks depict the intestine lumen, arrowheads indicate positive overlap. Scale bars: A, 10 μm; B, D, and F, 5 μm. Quantitative data are available in S1 File.
(TIF)

S8 Fig RNAi treatment of *zyg-12* has no effect on the tubular profile of hTAC-GFP. Scale bars: 10 μm.
(TIF)

S1 Table Mutant and transgenic strains used in this study. (DOCX)

S1 File This excel file contains the quantitative data used for panels presented in Figs 1–6, including supplementary Figs. (XLSX)
