# Supplementary figures and images for: SNX-3 mediates retromer-independent tubular endosomal recycling by opposing EEA-1-facilitated trafficking
Source: PLoS Genet. 2021 Jun 3;17(6):e1009607. doi: 10.1371/journal.pgen.1009607 (PMC8219167; doi:10.1371/journal.pgen.1009607)

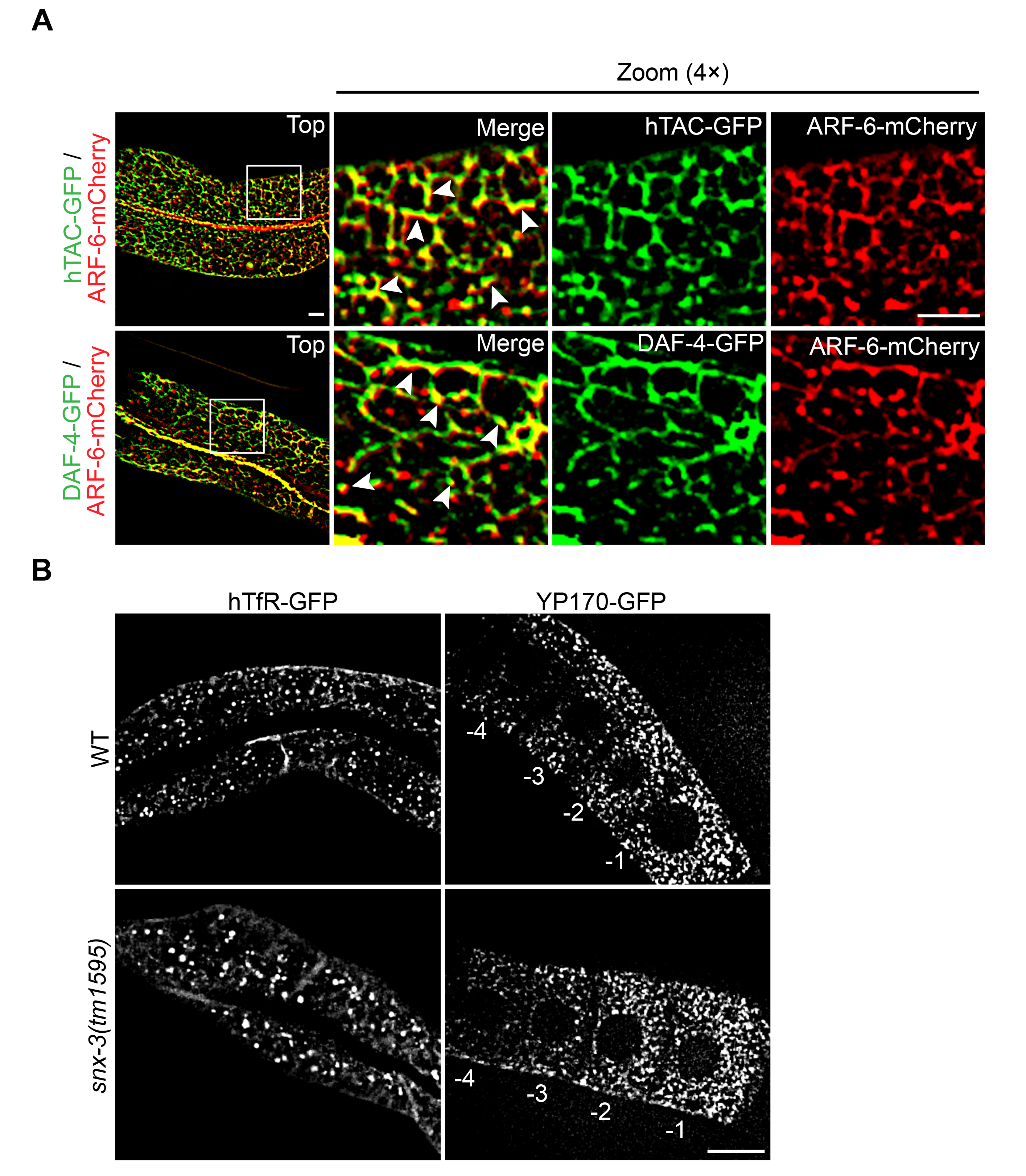

Supplement: S1 Fig — (A) Micrographs showing the subplasmalemmal hTAC-GFP or DAF-4-GFP tubules overlapped with ARF-6-mCherry-positive structures. The arrowheads indicate overlap. (B) Confocal images showing the steady-state distributions of two clathrin-dependent cargo proteins, the intestine-integrated hTfR-GFP and oocyte-internalized YP170-GFP, are comparable in snx-3(tm1595) mutants to those in WT animals. Oocytes proximal to the spermatheca are numbered as -1. Scale bars: A, 5 μm; B, 20 μm. (TIF) [file pgen.1009607.s001.tif]

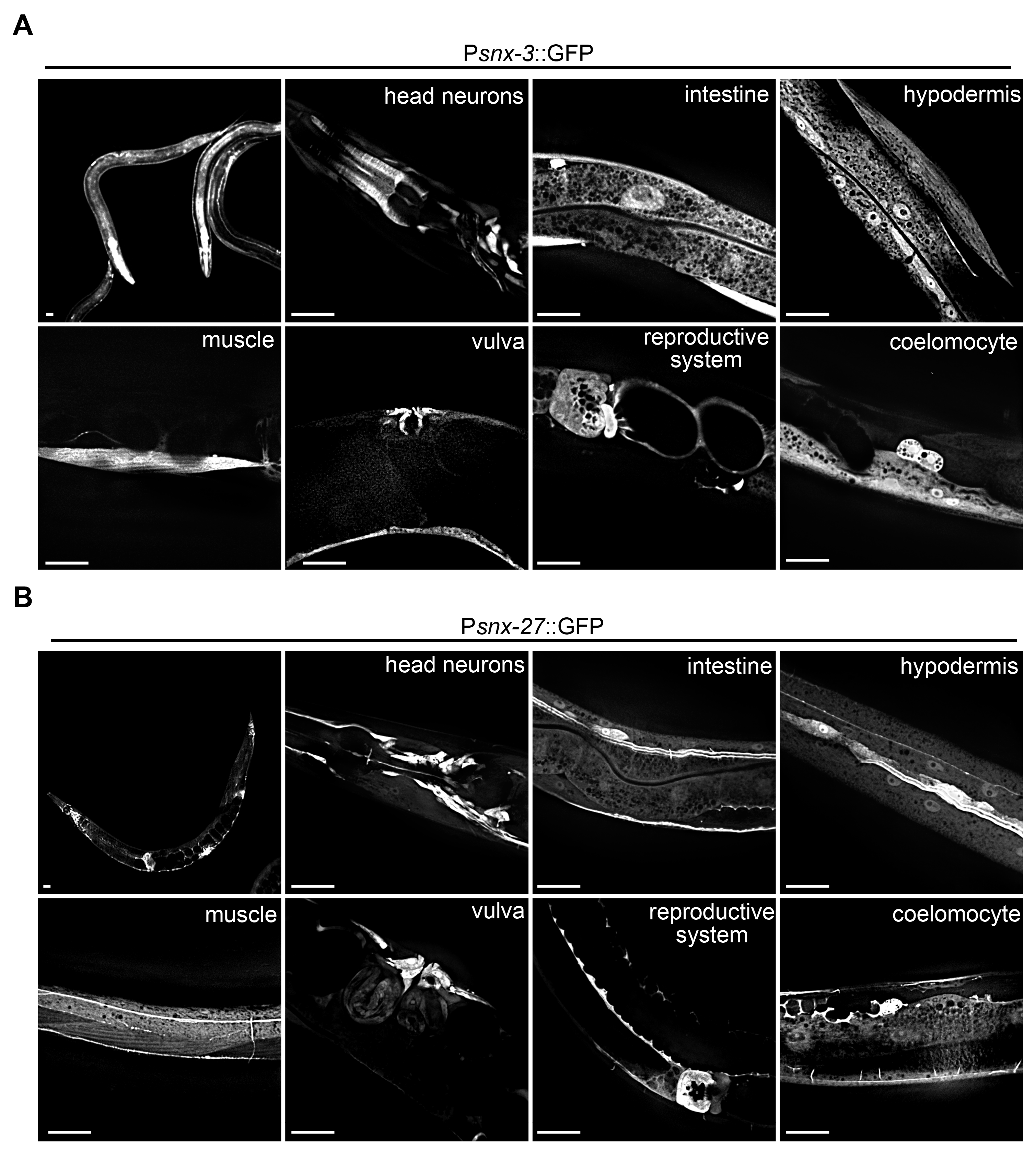

Supplement: S2 Fig — Confocal images of adult hermaphrodites expressing GFP driven by the snx-3 promoter (690 bp upstream of the start codon) (A) or the snx-27 promoter (about 5 kb upstream of the start codon) (B). GFP is broadly expressed in C. elegans, including head and tail neurons, intestine, hypodermis, muscle, vulva, reproductive system and coelomocytes. Scale bars: 20 μm. (TIF) [file pgen.1009607.s002.tif]

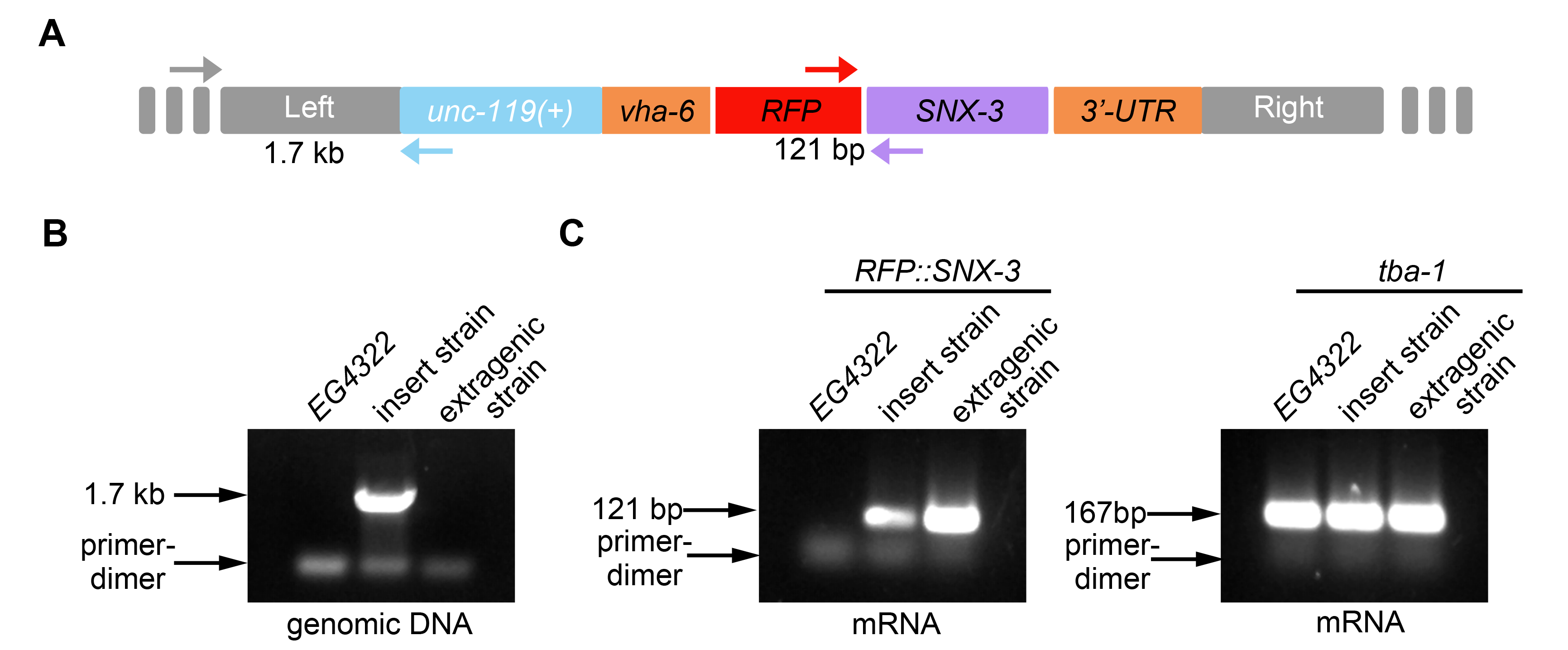

Supplement: S3 Fig — (A) Schematic of the targeting construct containing the unc-119(+) rescue gene and Pvha-6::RFP::SNX-3 transgene flanked by DNA homologous to the ttTi5605mos insertion site. (B) PCR verification of inserted Pvha-6::RFP::SNX-3 transgenes. The forward primer anneals to the genomic region outside of that contained in the targeting construct, and the reverse primer is in the unc-119(+) selectable marker. A PCR band of expected size (1.7 kb) was amplified only from the insertion strain. (C) PCR analysis of RFP::SNX-3 mRNA expression levels shows the mRNA level is lower in Pvha-6::RFP::SNX-3 transgene insertion strain than in Pvha-6::RFP::SNX-3 extragenic strain. The forward primer anneals to the C-terminal region of RFP, and the reverse primer anneals to the N-terminal region of SNX-3. (TIF) [file pgen.1009607.s003.tif]

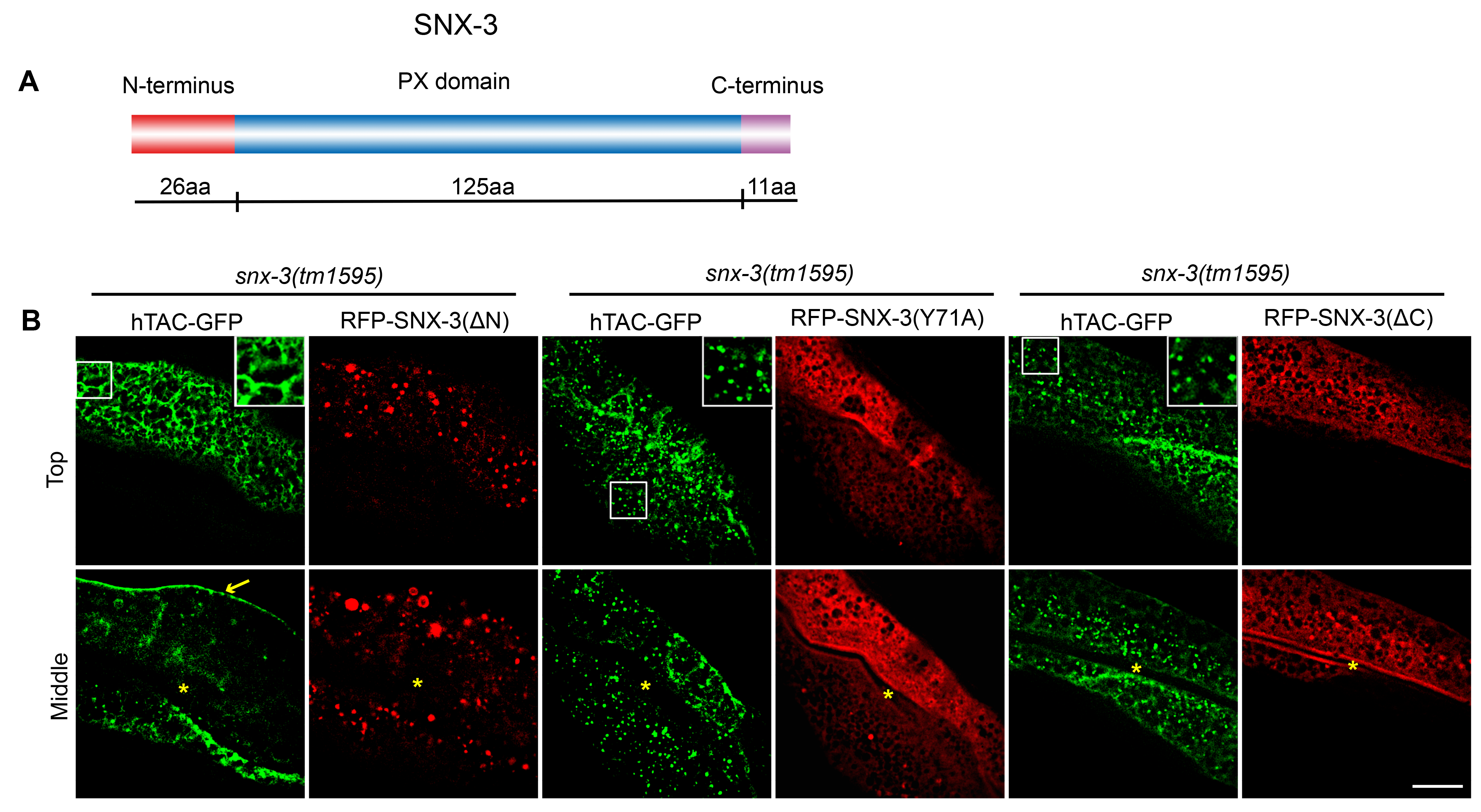

Supplement: S4 Fig — (A) Schematic diagram illustrating the domain architecture of SNX-3. (B) Confocal images show the fragment of N-terminus-deleted SNX-3 restore the tubular morphology of hTAC-containing structures in snx-3(tm1595) mutants, while the single point-mutated SNX-3(Y71A) or the fragment of C-terminus-deleted SNX-3 loses the capability. The Arrows indicate basolateral PM-associated hTAC-GFP. Asterisks depict the intestine lumen. Scale bar: 10 μm. (TIF) [file pgen.1009607.s004.tif]

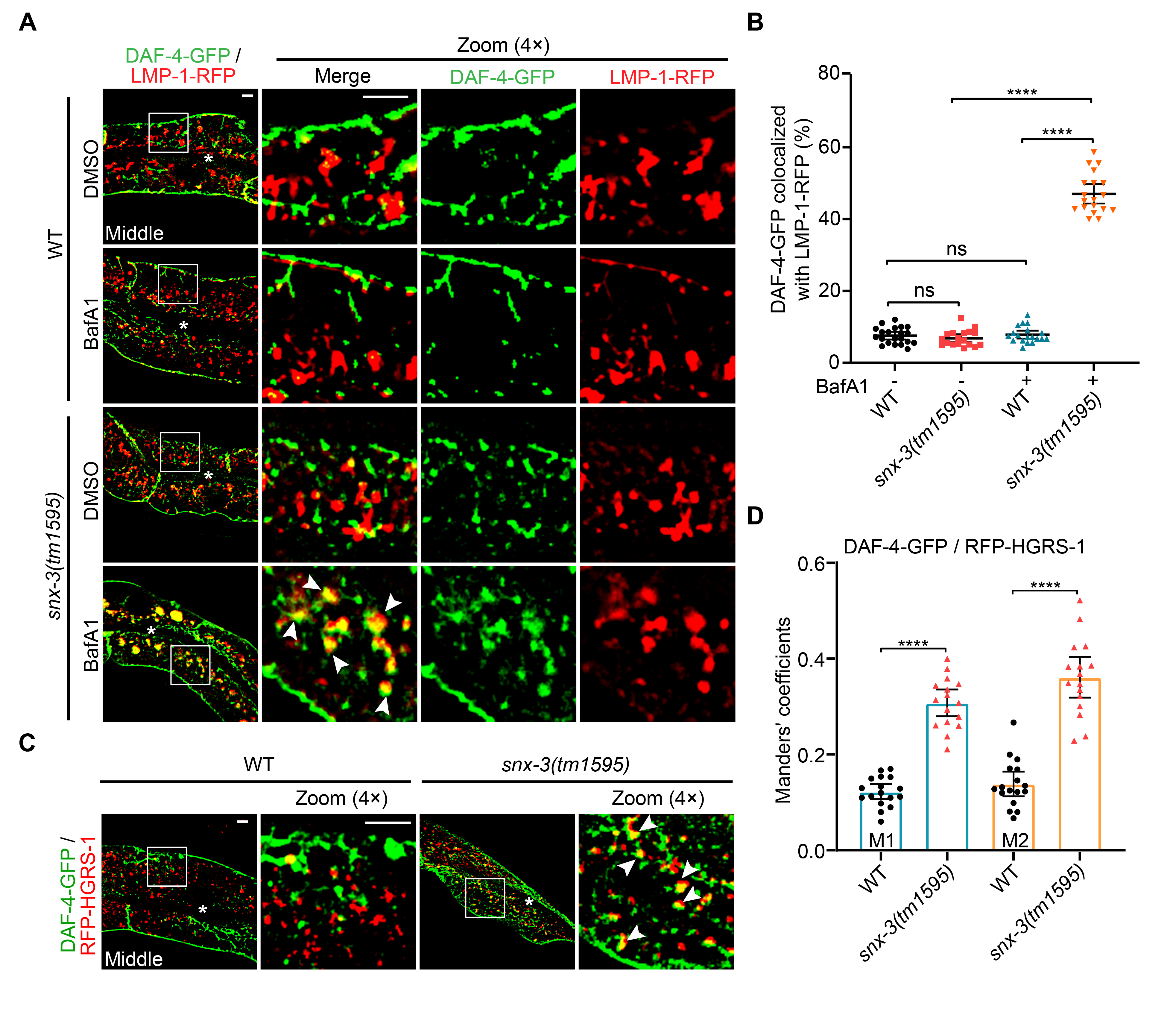

Supplement: S5 Fig — (A) Micrographs showing the colocalization of DAF-4-GFP with LMP-1-RFP in snx-3(tm1595) mutants pretreated with BafA1. In WT animals, no obvious colocalization of DAF-4-GFP and LMP-1-RFP signals is observed either in the absence (7.5%) or presence (7.8%) of BafA1. By contrast, BafA1 treatment leads to accumulation of DAF-4-GFP that prominently overlapped with LMP-1-RFP signals in snx-3(tm1595) mutants (47.1%), indicating DAF-4 is efficiently degraded in the lysosome of snx-3(tm1595) mutants. (B) Colocalization of DAF-4-GFP with LMP-1-RFP was calculated for each condition as depicted in A. [ROI] = 21/18/19/19, n = 13/10/14/10 for WT(-)/snx-3(tm1595)(-)/WT(+)/snx-3(tm1595)(+). Error bars are mean ± 95% CI. ns, not significant; ****P<0.0001(Brown-Forsythe and Welch ANOVA with Dunnett T3 multiple comparison test). (C) Confocal images showing the overlap of DAF-4-GFP with RFP-HGRS-1 is increased in snx-3(tm1595) mutants. (D) Manders’ coefficients for DAF-4-GFP and RFP-HGRS-1 as depicted in C were calculated, M1 = 0.12 vs. 0.30, M2 = 0.14 vs. 0.36, for WT vs. snx-3(tm1595) mutant animals, error bars are mean ± 95% CI (WT: [ROI] = 17, n = 9; snx-3(tm1595): [ROI] = 16, n = 10). ****P<0.0001 (t test with Welch’s correction for M1; Student’s t test for M2). M1: green pixels overlapping red; M2: red pixels overlapping green. In A and C, asterisks depict the intestine lumen, arrowheads indicate positive overlap. Scale bars: 5 μm. Quantitative data are available in S1 File. (TIF) [file pgen.1009607.s005.tif]

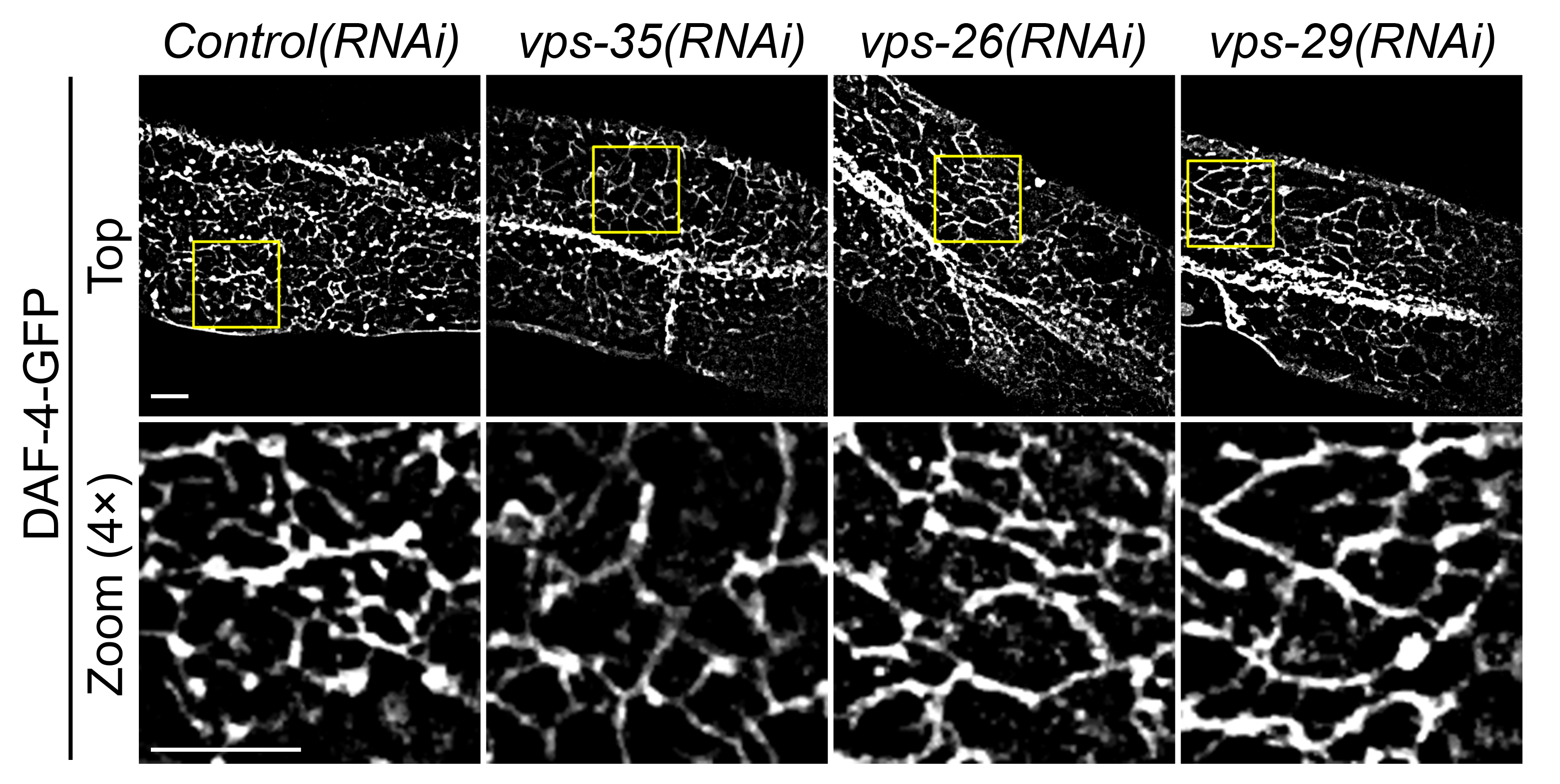

Supplement: S6 Fig — The tubular profile of subplasmalemmal DAF-4-GFP-containing carriers is not changed upon RNAi treatment of vps-35, vps-26, or vps-29. Scale bars: 5 μm. (TIF) [file pgen.1009607.s006.tif]

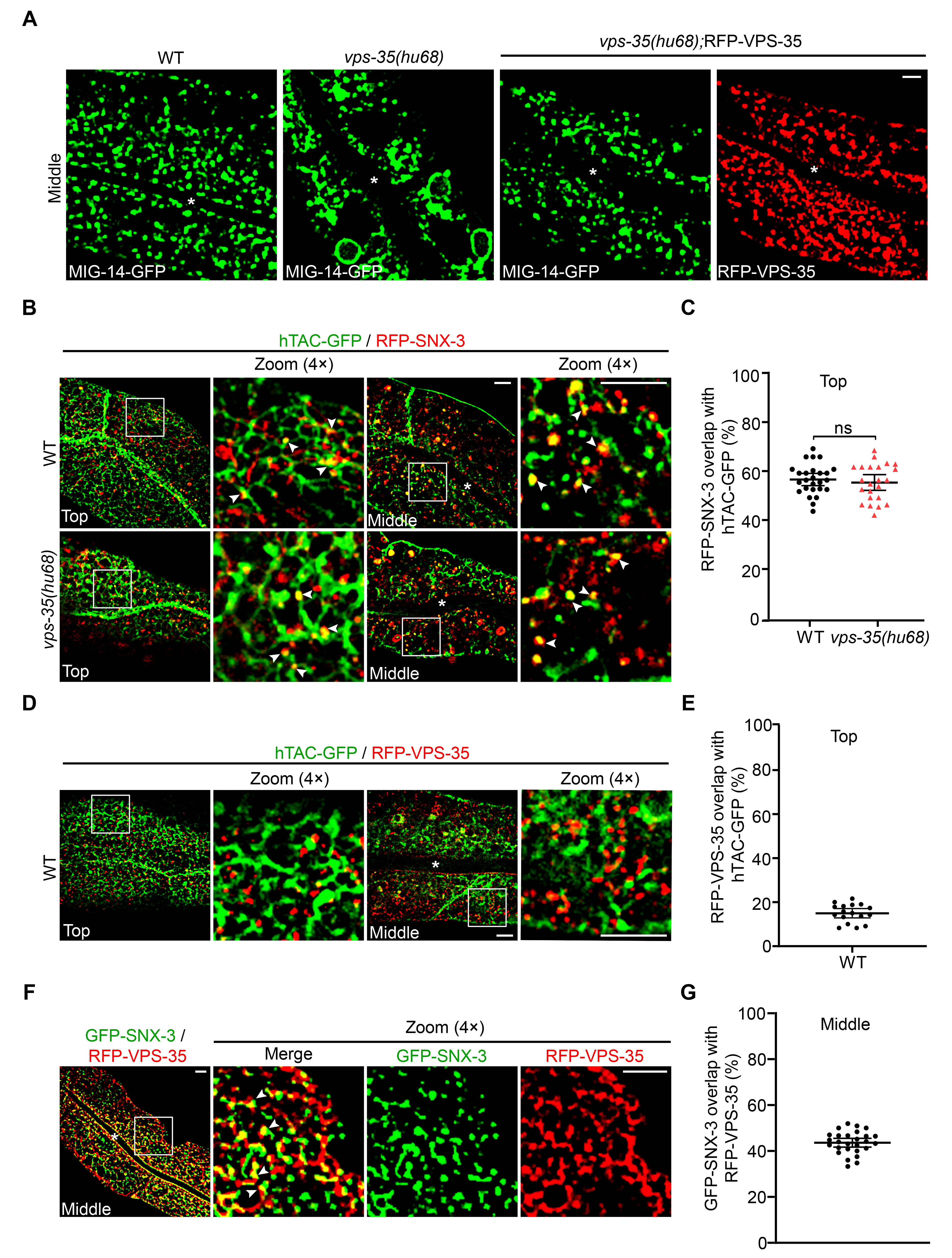

Supplement: S7 Fig — (A) Confocal images show the transgene of vha-6 driven RFP-VPS-35 restore the punctate morphology of MIG-14-GFP in vps-35(hu68) mutant animals, indicating the RFP-tagged VPS-35 is functional. (B) Micrographs showing the localization of RFP-SNX-3 at the tips of hTAC-GFP tubules in WT and vps-35(hu68) mutant animals. (C) Percentage of RFP-SNX-3 overlapped with hTAC-GFP as depicted in B was calculated, error bars are mean ± 95% CI (WT: [ROI] = 26, n = 9; vps-35(hu68): [ROI] = 23, n = 9). ns, not significant (Student’s t test). (D) Micrographs showing very few RFP-VPS-35 locate at hTAC-containing structures. (E) Percentage of RFP-VPS-35 overlapped with hTAC-GFP as depicted in D was calculated, error bars are mean ± 95% CI ([ROI] = 17, n = 4). (F) Micrographs showing partial overlap of co-expressed GFP-SNX-3 and RFP-VPS-35 in the C. elegans intestine. (G) Percentage of GFP-SNX-3 overlapped with RFP-VPS-35 as depicted in F was calculated, error bars are mean ± 95% CI ([ROI] = 27, n = 21). In A, B, D, and F, asterisks depict the intestine lumen, arrowheads indicate positive overlap. Scale bars: A, 10 μm; B, D, and F, 5 μm. Quantitative data are available in S1 File. (TIF) [file pgen.1009607.s007.tif]

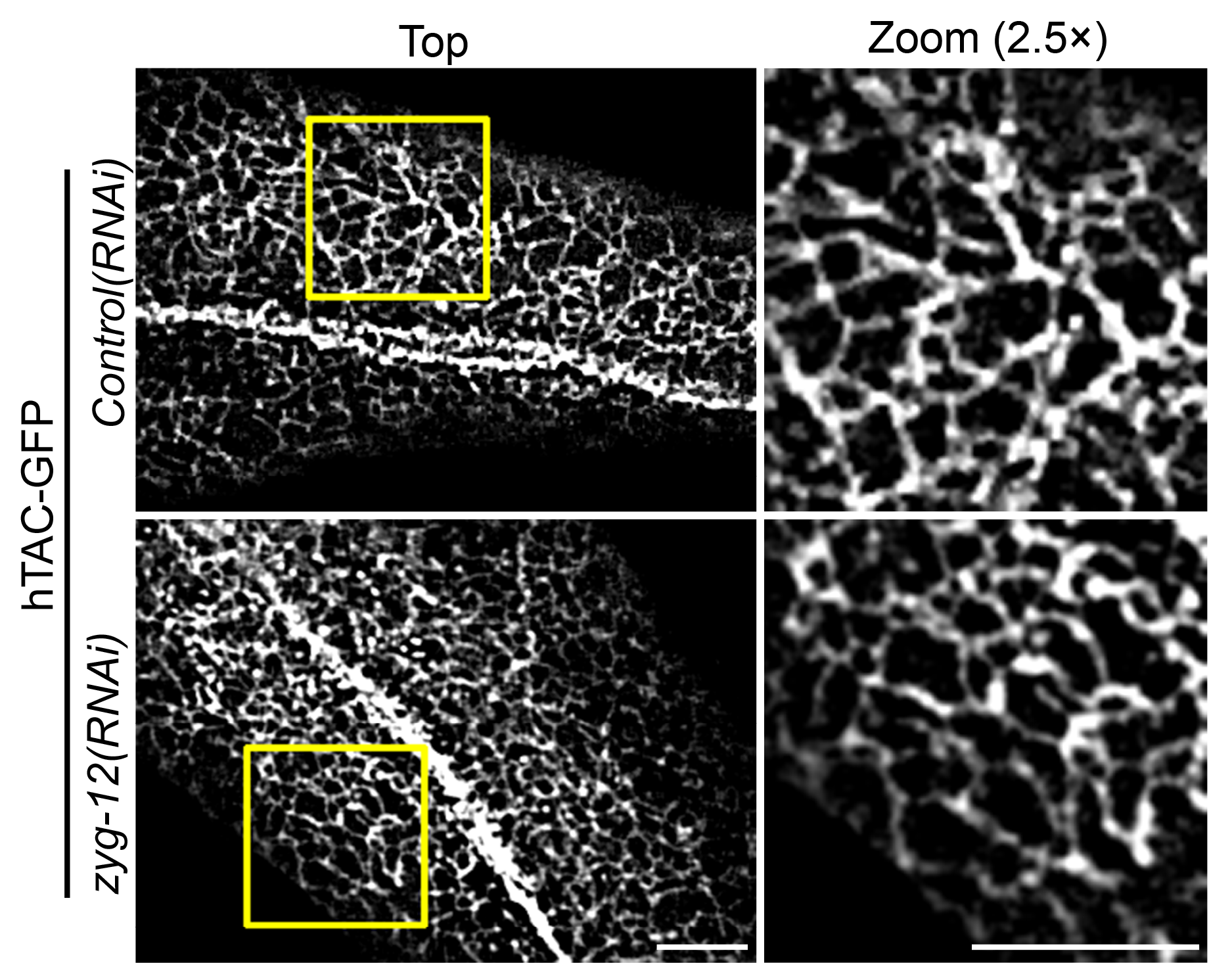

Supplement: S8 Fig — Scale bars: 10 μm. (TIF) [file pgen.1009607.s008.tif]
