# Supplementary material for: SNX-3 mediates retromer-independent tubular endosomal recycling by opposing EEA-1-facilitated trafficking
Source: PLoS Genet. 2021 Jun 3;17(6):e1009607. doi: 10.1371/journal.pgen.1009607 (PMC8219167; doi:10.1371/journal.pgen.1009607)
Supplement: S1 Table — (DOCX) [file pgen.1009607.s009.docx]

**S1 Table. Mutant and transgenic strains used in this study.**

| **Strain** | **Genotype** |
| --- | --- |
| *tm847* | *snx-1(tm847) X ^a^* |
| *tm1595* | *snx-3(tm1595) I ^a^* |
| *tm2404* | *snx-13(tm2404) IV ^a^* |
| *tm3643* | *snx-17(tm3643) X ^a^* |
| *tm5356* | *snx-27(tm5356) I ^a^* |
| *tm2423* | *lst-4(tm2423) IV ^a^* |
| KN555 | *vps-35(hu68) II ^e^* |
| GS2477 | *arIs37;cup-5(ar465)Ⅲ;dyp-20(e1282) IV ^b^* |
| *tm1523* | *vps-26(tm1523) IV ^a^* |
| *tm1320* | *vps-29(tm1320) III ^a^* |
| RT393 | *unc-119(ed3) III; pwIs112 [Pvha-6::hTAC-GFP;Cbr-unc-119(+)] ^c^* |
| RT1970 | *unc-119(ed3) III; pwIs90[Pvha-6::hTfR::GFP;Cbr-unc-119(+)] ^c^* |
| RT2495 | *unc-119(ed3) III; pwIs922[Pvha-6::DAF-4::GFP;Cbr-unc-119(+)] ^c^* |
| RT548 | *unc-119(ed3) III; pwIs216[Pvha-6::RFP::RME-1;Cbr-unc-119(+)] ^c^* |
| RT2071 | *unc-119(ed3) III; pwIs765[Pvha-6::MIG-14::GFP;Cbr-unc-119(+)]* ^c^ |
| DH1006 | *bIs1* *[Pvit-2::VIT-2::GFP;rol-6(su1006)] ^c^* |
|  | *pwIs518 [Pvha-6::GFP::HGRS-1] ^e^* |
| EG4322 | *ttTi5605 II; unc-119(ed3) III ^f^* |
|  | *ryzIs1 [Pvha-6::TagRFP-T::SNX-3]* |
| RYZEx23 | *[Psnx-3::GFP] ^d^* |
| RYZEx25 | *[Psnx-27::GFP] ^d^* |
| RYZEx26 | *[Pvha-6::TagRFP-T::SNX-3(del N)] ^d^* |
| RYZEx27 | *[Pvha-6::TagRFP-T::SNX-3(del C)] ^d^* |
| RYZEx28 | *[Pvha-6::TagRFP-T::SNX-3(Y71A)] ^d^* |
| RYZEx30 | *[Pvha-6::TagRFP-T::SNX-3] ^d^* |
| txuEx14 | *[Pvha-6::TagRFP-T::RAB-5] ^d^* |
| RYZEx19 | *[Pvha-6::GFP::EEA-1] ^d^* |
| RYZEx20 | *[Pvha-6::TagRFP-T::EEA-1] ^d^* |
| RYZEx29 | *[Pvha6::LMP-1::TagRFP-T] ^d^* |
| RYZEx21 | *[Pvha-6::TagRFP-T::VPS-35] ^d^* |
| RYZEx18 | *[Pvha-6::TagRFP-T::HGRS-1] ^d^* |
| RYZEx33 | *[Pvha6::ARF-6::mCherry] ^d^* |
| RYZEx34 | *[Pvha-6::GFP::SNX-3] ^d^* |

a. These mutant strains were provided by the National BioResource Project (Tokyo Women’s Medical University, Tokyo, Japan). b. This strain was provided by the Caenorhabditis Genetics Center (University of Minnesota, Minneapolis, MN). c. These integrated transgenic strains were gifts from Professor Barth D. Grant (Rutgers University, Piscataway, NJ, USA). d. These extrachromosomal transgenic strains were obtained by using standard microinjection techniques. e. This strain was gift from Professor Anbing Shi (Huazhong University of Science and Technology, Wuhan, China). f. This strain was gift from Professor Erik M. Jorgensen (University of Utah, Salt Lake City).
